# Short-Term Synaptic Plasticity Makes Neurons Sensitive to the Distribution of Presynaptic Population Firing Rates

**DOI:** 10.1523/ENEURO.0297-20.2021

**Published:** 2021-04-08

**Authors:** Luiz Tauffer, Arvind Kumar

**Affiliations:** 1 Department of Computational Science and Technology, School of Computer Science and Communication, KTH Royal Institute of Technology, 11428 Stockholm, Sweden; 2 Bernstein Center Freiburg, University of Freiburg, 79104 Freiburg im Breisgau, Germany

**Keywords:** excitation/inhibition balance, neural code, short-term plasticity, sparse code, synaptic depression, synaptic facilitation

## Abstract

The ability to discriminate spikes that encode a particular stimulus from spikes produced by background activity is essential for reliable information processing in the brain. We describe how synaptic short-term plasticity (STP) modulates the output of presynaptic populations as a function of the distribution of the spiking activity and find a strong relationship between STP features and sparseness of the population code, which could solve this problem. Furthermore, we show that feedforward excitation followed by inhibition (FF-EI), combined with target-dependent STP, promote substantial increase in the signal gain even for considerable deviations from the optimal conditions, granting robustness to this mechanism. A simulated neuron driven by a spiking FF-EI network is reliably modulated as predicted by a rate analysis and inherits the ability to differentiate sparse signals from dense background activity changes of the same magnitude, even at very low signal-to-noise conditions. We propose that the STP-based distribution discrimination is likely a latent function in several regions such as the cerebellum and the hippocampus.

## Visual Abstract

**Figure:**
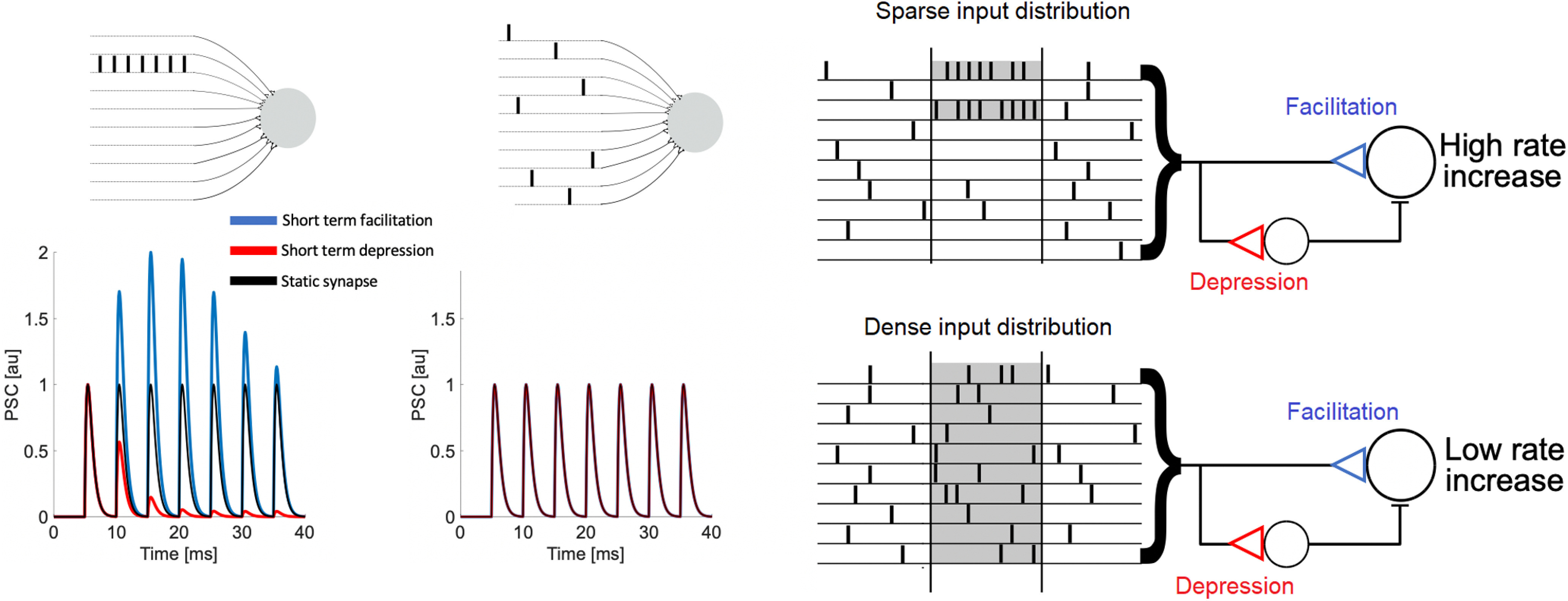

## Significance Statement

What is the optimal way to distribute a fixed number of spikes over a set of neurons so the we get a maximal response in the downstream neuron? This question is at the core of neural coding. Here, we show that when synapses show short-term facilitation, sparse code (when a few neurons increase their firing rate in a task-dependent manner) is more effective than dense code (when many neurons increase their firing rate in a task-dependent manner). By contrast, when synapses show short-term depression a dense code is more effective than a sparse code. Thus, for the first time, we show that the dynamics of synapses itself has an effect in deciding the most effective neural code

## Introduction

The brain is a highly noisy system. At the cellular level, the neurons are unreliable in eliciting spikes and synapses are unreliable in transmitting the spikes to the postsynaptic neurons. At the network level, the connectivity and balance of excitation and inhibition gives rise to fluctuations in the background activity (Brunel, 2000; Kumar et al., 2008), which can be as high as the mean stimulus response (Arieli et al., 1996; Kenet et al., 2003). In such a noisy environment, a neuron is faced with a crucial task: how to discriminate stimulus-induced firing rate changes from fluctuations in the firing rate of the background activity of the same magnitude?

If synapses were static, that is, when the postsynaptic conductances (PSCs) do not depend on the immediate spike history, this task could not be accomplished, unless synapses are specifically tuned to do so. For instance, the identification of specific spiking patterns, filtering out presumed noise sequences, can be accomplished by precise tuning of synaptic weights (Gütig and Sompolinsky, 2006). This solution, however, relies on training synaptic weights using a certain supervised learning rule, and even then, it could only work for a specific set of spike timing sequences. Active dendrites (with voltage dependent ionic conductance) can also work as pattern detectors (Hawkins and Ahmad, 2016), but this mechanism would only work for signals constrained to locally clustered synapses. Therefore, despite being relevant for the understanding of signal processing in the brain, the mechanisms by which neural ensembles solve the activity discrimination problem have remained elusive.

Here, we show that short-term plasticity (STP) of synapses provides an effective and general mechanism to solve the aforementioned task. STP refers to the observation that synaptic strength changes on spike-by-spike basis, depending on the timing of previous spikes (Stevens and Wang, 1995; Zucker and Regehr, 2002), that is, STP arises because neurotransmitter release dynamics is history dependent and can be manifest as either short-term facilitation (STF) or short-term depression (STD). Thus, STP becomes a crucial part of neural hardware when information is encoded as firing rate. Indeed, STP has been suggested to play several important roles in neural information processing (Buonomano, 2000; Fuhrmann et al., 2002; Izhikevich et al., 2003; Abbott and Regehr, 2004; Middleton et al., 2011; Rotman et al., 2011; Scott et al., 2012; Rotman and Klyachko, 2013; Jackman and Regehr, 2017; Grangeray-Vilmint et al., 2018; Naud and Sprekeler, 2018).

**Figure 1.**
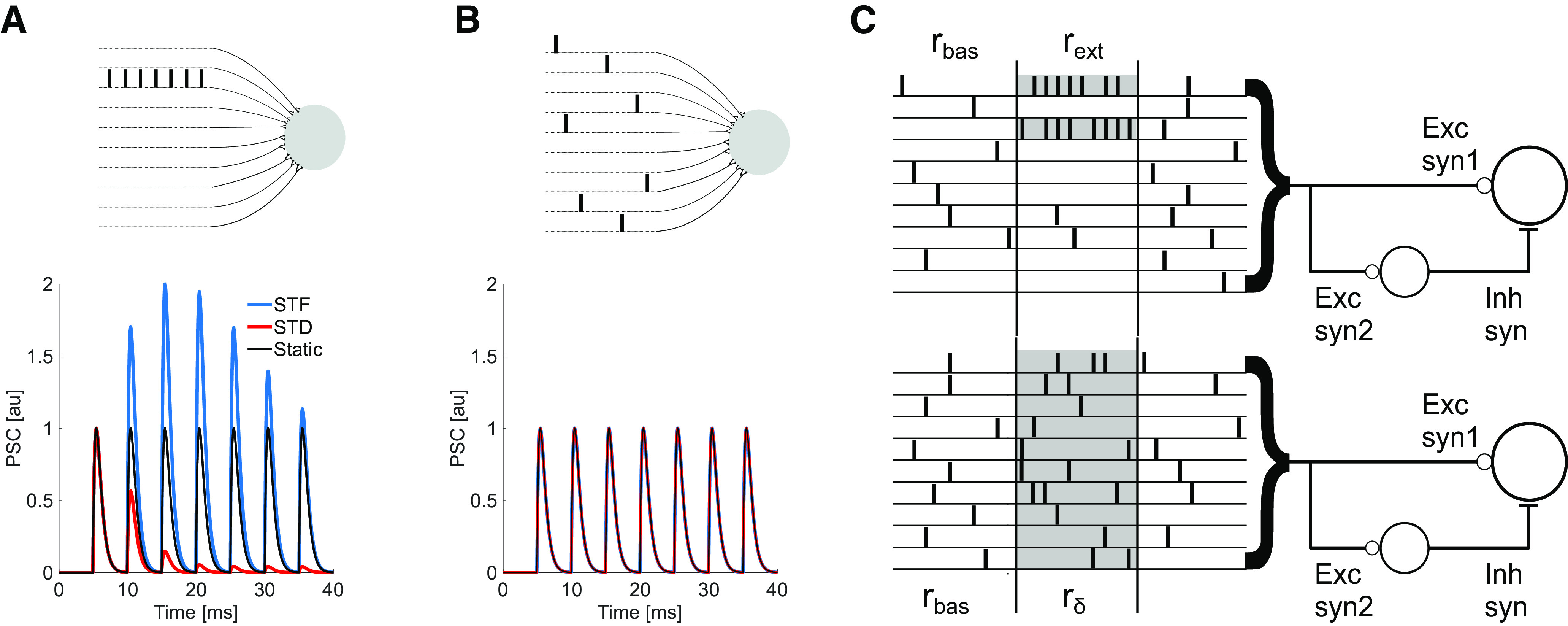
Distribution of the spiking activity over presynaptic neurons and STP. ***A***, top, A neuron receives input from a presynaptic population, with only one of the neurons eliciting seven spikes. Bottom, The postsynaptic conductance (PSC) generated by the consecutive spikes for three different types of synapses (static, black; facilitatory, blue; depressing, red). The PSCs are different for each of these three types of synapses. ***B***, top, Similar to panel ***A***, a neuron receives inputs from a presynaptic population, but in this scenario the spikes were distributed among all presynaptic neurons. Bottom, The PSC generated by a sequence of seven consecutive spikes arriving at the same time as in panel ***A*** coming from three different types of synapses (static, black; facilitatory, blue; depressing, red). The PSCs are identical for each of these three types of synapses (lines overlapped). ***C***, Feedforward excitation followed by feedforward inhibition configuration and two distributions of an extra spike rate *R_ext_* in addition to the basal firing rate *r_bas_*. Top, The extra rate is distributed into a few presynaptic neurons *N_ext_* units (gray), with each chosen unit increasing its rate by $r_{ext}=R_{ext}/N_{ext}$. Bottom, The extra rate is distributed homogeneously throughout the population of *N* units, with each unit increasing its rate by $r_{\delta }=R_{ext}/N$.

An immediate consequence of STP is that the effective PSCs depend on the firing rates of individual presynaptic neurons (Fig. 1). This suggests that postsynaptic targets of populations with dynamic synapses could distinguish among different input firing rate distributions even without supervised learning. To demonstrate this feature of STP, we measured the response of postsynaptic neurons for a weak stimulus with amplitude one order of magnitude smaller than the background activity. By systematically changing the distribution of firing rates over the presynaptic neuron ensemble, we found that weak signals can be differentiated from the noisy fluctuations if the signal is appropriately distributed over the input ensemble. The optimal distribution that maximizes the discriminability depends on the nature of STP. We found that, for facilitatory synapses, sparse codes give better discrimination between a weak signal and dense background changes of the same intensity. By contrast, for depressing synapses, sparse codes result in highly negative gains in relation to dense background changes of the same magnitude. We also investigated feedforward networks with excitation and disynaptic inhibition, with target-dependent STP, and found that this arrangement allows for extra robustness for the output gain.

Finally, we demonstrate how STP can endow a postsynaptic neuron with the ability to differentiate sparsely encoded activity from dense activity of the same magnitude, a function that would be especially important at very low signal-to-noise regimes. Thus, our results reveal that the nature of STP may also constrain the nature of firing rate-based population code.

## Materials and Methods

### Model of STP

One parsimonious and yet powerful mathematical description of short-term synaptic dynamics was proposed already 20 years ago (Tsodyks and Markram, 1997). The Tsodyks–Markram (TM) model could first account for activity-dependent synaptic depression observed in pairs of neocortical pyramidal neurons and was soon extended to cover for facilitation (increase in probability) of vesicle release (Tsodyks et al., 1998). With a small set of parameters, the TM model is able to explain the opposed effects of depletion of available synaptic vesicles and of the increase in release probability caused by accumulation of residual calcium in the presynaptic terminal, making it suitable as a framework to conjecture general impact of STP in neural information processing.

Here, we use the TM model (Eq. 1) to describe the short-term synaptic dynamics. The effect of depression is modeled by depletion of the proportion of available resources, represented by the variable *x* (0 ≤ *x *≤* *1), which instantaneously decreases after each spike and returns to 1 with recovery time *τ_rec_*. The gain effect of short-term facilitation is modeled by the facilitation factor *U* (0 ≤ *U *≤* *1), which accounts for the accumulation of calcium at the presynaptic terminal after the arrival of an action potential. *U* transiently increases the release probability *u* (0 ≤ *u *≤* *1), which returns to 0 with time constant *τ_f_*:

(1) $$\begin{array}{l} \frac{du^{-}}{dt}=-\frac{u^{-}}{\tau_{f}}+U(1-u^{-})\delta (t-t_{sp})\\ u^{+}=u^{-}+U(1-u^{-})\\ \frac{dx}{dt}=\frac{1-x}{\tau_{rec}}-u^{+}x^{-}\delta (t-t_{sp})\\ \frac{dg^{s}}{dt}\propto B^{s}u^{+}(t_{sp})x^{-}(t_{sp}) \end{array},$$

where *t_sp_* is the last spike time.

### Proportion of released resources (*PRR*)

The change in the PSC *g^s^* after a presynaptic spike is proportional to the instantaneous *PRR* ($PRR(t_{sp})\propto u^{+}(t_{sp})x^{-}(t_{sp})$) and to the absolute synaptic strength *B^s^*. The average instantaneous *PRR* of a presynaptic unit can also be described as a function of a time-dependent Poissonian firing rate *r*(*t*) (Tsodyks et al., 1998) as:

(2) $$\begin{array}{l} \frac{d〈u〉}{dt}=-\frac{〈u〉}{\tau_{f}}+U(1-〈u^{-}〉)r(t)\\ 〈u^{+}〉=〈u^{-}〉+U(1-〈u^{-}〉)\\ \frac{d〈x〉}{dt}=\frac{1-〈x〉}{\tau_{rec}}-〈u^{+}〉〈x^{-}〉r(t)\\ PRR^{s}(t)=〈u^{+}〉〈x^{-}〉r(t), \end{array}$$

where the brackets denote the average over many realizations. The total *PRR* contribution of a single synapse, for a time window of duration *T_s_*, can then be obtained by integrating Equation 2 over this period:

(3) $$Q^{s}=\int_{0}^{T_{s}}PRR^{s}(t)dt.$$

### Total effective input to a postsynaptic neuron

For a homogeneous presynaptic population with same STP parameters and individual basal firing rate *r_bas_*, the population basal activity is $R_{bas}=N\cdot r_{bas}$, where *N* is the population size. We quantify *R_ext_* as a multiple of *R_bas_*. Our analysis is restricted to the case of low signal-to-noise ratio, i.e., $R_{ext}<0.1R_{bas}$. We consider a simplified scenario where *R_ext_* is distributed homogeneously through a number *N_ext_* of selected presynaptic units, which will increase their firing rate by $r_{ext}=R_{ext}/N_{ext}$, while the remaining presynaptic units will keep their activity unchanged.

The total *PRR* released to a target neuron by the entire population, during *T_s_*, will then be

(4) $$Q_{ext}^{p}=(N-N_{ext})\cdot Q_{bas}^{s}+N_{ext}\cdot Q_{ext}^{s},$$

where $Q_{bas}^{s}$ and $Q_{ext}^{s}$ are the total *PRR* (Eq. 3) delivered by a stationary unit (firing at *r_bas_*) and a stimulus encoding unit (firing at *r_bas_* + *r_ext_*), respectively.

### Gain in the effective input

We are interested in the effects of varying the presynaptic distribution (over *N_ext_* inputs) of this total extra rate (*R_ext_*) on the effective input to postsynaptic targets. To estimate the change in the gain because of STP we used the maximally dense distribution, when *N_ext_* = *N* as the reference point:

(5) $$Q_{\delta }^{p}=N\cdot Q_{\delta }^{s},$$

where the *δ* subscript denotes the smallest possible increase in individual firing rates, *r_δ_* (maximally distributed *R_ext_*). We refer to this as the dense distribution case and it ideally represents a situation of homogeneous increase in the basal activity of the system, against which a stimulus would need to be distinguished from. *N_ext_* = *N* also implies smallest increase in individual operating rates (*r_ext_* = *r_δ_*), therefore in the dense distribution case STP nonlinearities will be minimal. In other words, *N_ext_* = *N* is the point where dynamic synapses will operate as close to static as possible.

We then quantify the gain in $Q_{ext}^{p}$ for a given *N_ext_* always relative to $Q_{\delta }^{p}$ caused by an input of the same intensity but with dense distribution, as

(6) $$G=100\cdot (\frac{Q_{ext}^{p}-Q_{bas}^{p}}{Q_{\delta }^{p}-Q_{bas}^{p}}-1).$$

**Figure 2.**
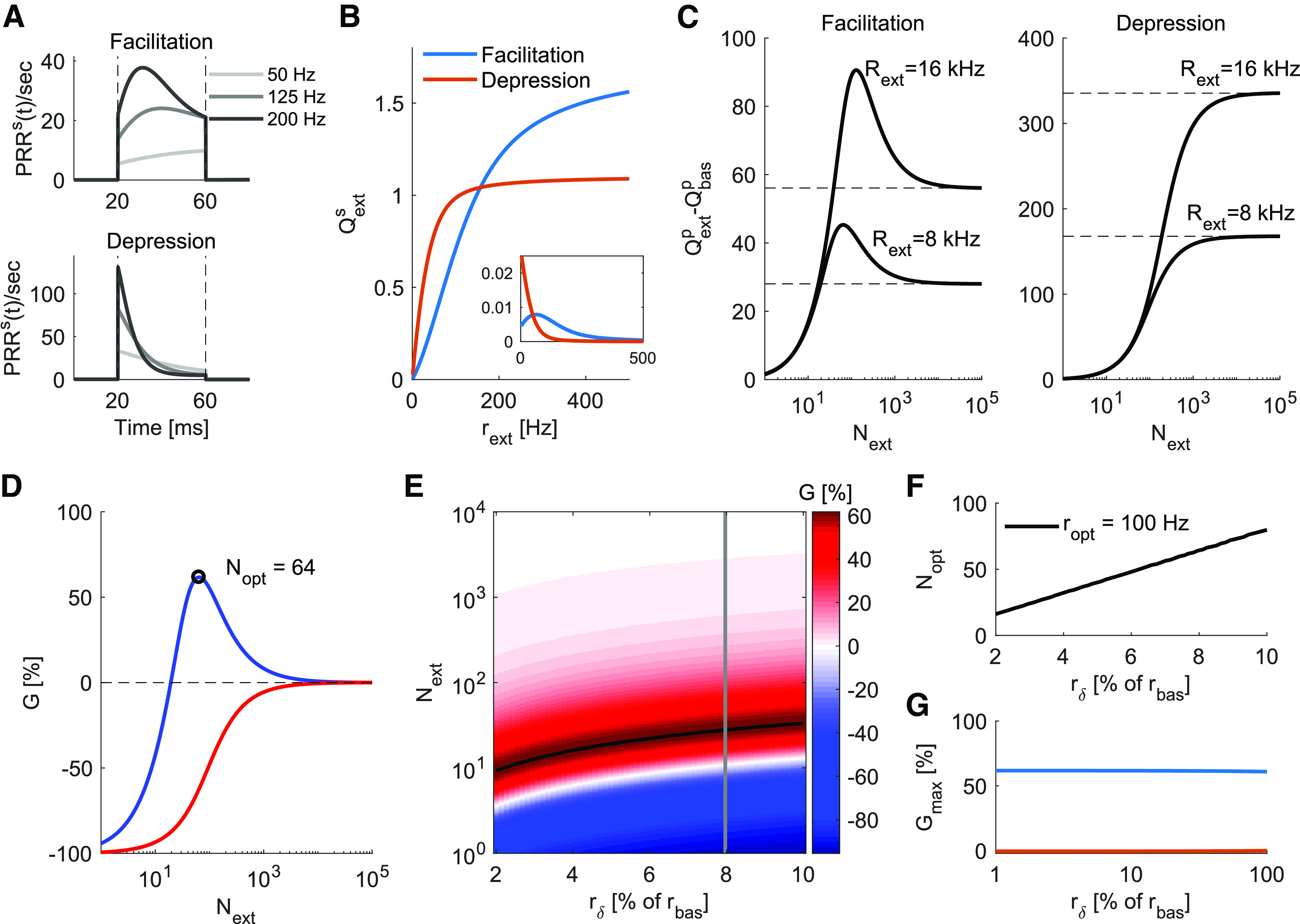
***A***, Temporal profile of the *PRR* for a facilitatory synapse (top, *U *=* *0.1, $\tau_{f}=200\;\text{ms}$ and $\tau_{rec}=50\;\text{ms}$) and a depressing synapse (bottom, *U *=* *0.7, $\tau_{f}=50ms$ and $\tau_{rec}=200\;\text{ms}$), with increased rates during a period of $T_{s}=40\;\text{ms}$. ***B***, The amount of resources released by a single synapse, *Q^s^*. This was obtained by integrating $PRR^{s}(t)$ over *T_s_* (area under the curve in ***A***, Eq. 3). $Q_{ext}^{s}$ for depressing synapses saturates at lower firing rates than facilitatory synapses. Inset, The derivative of $Q_{ext}^{s}$ and highlights the nonlinearities in $Q_{ext}^{s}$, with depressing synapses showing monotonically decreasing slopes (decreasing release rate) and facilitatory synapses showing an initial region of increasing slopes (increasing release rate) with respect to *r_ext_*. ***C***, The extra *PRR* ($Q_{ext}^{p}-Q_{bas}^{p}$) as a function of the number of presynaptic neurons (*N_ext_*) whose firing rate increases by two different values of *R_ext_*. Dashed lines mark the value achieved when $N_{ext}=N$, i.e., the dense distribution case ($Q_{\delta }^{p}-Q_{bas}^{p}$). A population of facilitatory synapses (left) maximizes its release with low *N_ext_*, while a population of depressing synapses (right) maximizes its release with $N_{ext}=N$. ***D***, The gain (*G*; Eq. 5) as a function of *N_ext_* for a fixed *R_ext_*. The *N_ext_* that maximizes *G*, for this particular extra rate, is *N_opt_* = 64 for facilitatory synapses and $N_{opt}=N$ for depressing synapses. Notice that if the extra rate is allocated in even fewer input units, *G* can be negative. ***E***, *G* surface for a facilitatory synapse as a function of $r_{\delta }$ and *N_ext_*. The black line marks the maximum values of *G*, i.e., *N_opt_* for each $r_{\delta }$. The gain curves at panel ***D***, where $r_{\delta }=8\%$ of *r_bas_*, is marked with a gray line for reference. ***F***, The relationship between *N_opt_* and $r_{\delta }$ is linear. At maximum gain (*G_max_*), the firing rate of the event-related neurons (*N_ext_*) is $r_{opt}(100Hz$ for this specific example). ***G***, *G_max_* for the two STP regimes shown in panel ***B***. For a low signal-to-basal ratio $r_{\delta }/r_{bas}<1$, the gain can be considered independent from the stimulus intensity $r_{\delta }$.

We calculate the curves of *G* as a function of *N_ext_* for different sets of STP parameters and basal rates and search for points where it is maximized (*N_ext_*= *N_opt_*), which we call the optimal distribution (see example in Fig. 2*D*).

### Optimal distribution

The optimal distribution of the activity (*OD*) can be framed as the fraction of the optimal number of encoding units *N_opt_* in a given population of size *N*, that is, $OD=N_{opt}/N$. Because the optimal code ($N_{opt}=R_{ext}/r_{opt}$) is the distribution that maximizes the gain over the dense distribution with the same input magnitude ($N=R_{ext}/r_{\delta }$), *OD* can be written as

(7) $$\begin{array}{ll} OD & =\frac{N_{opt}}{N}\\ & =\frac{R_{ext}}{r_{opt}}\cdot \frac{r_{\delta }}{R_{ext}}\\ & =\frac{r_{\delta }}{r_{opt}}. \end{array}$$

**Figure 3.**
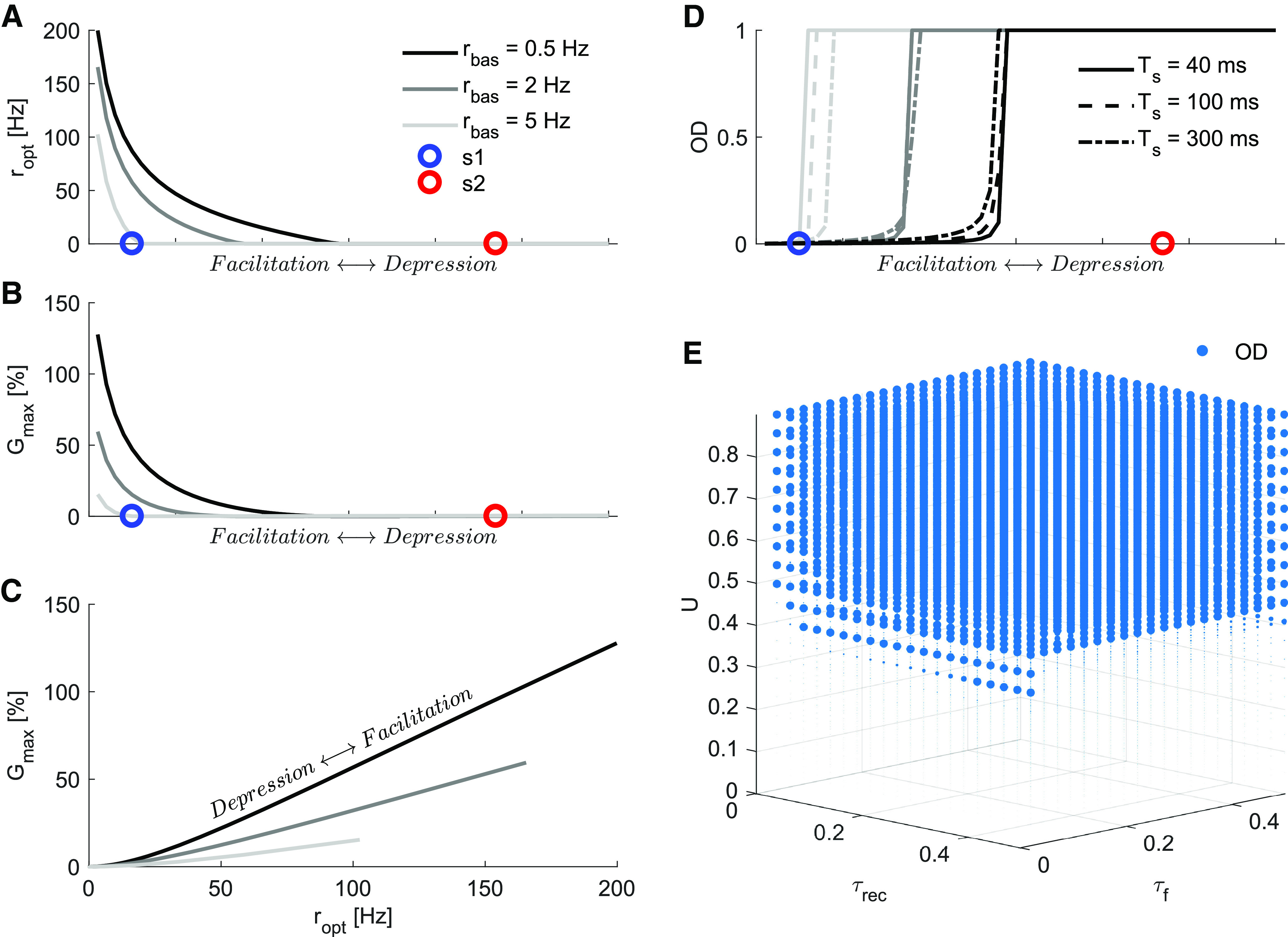
Effects of STP attributes on maximum gain of the neural population. Here, the STP parameters *U*, *τ_rec_* and *τ_f_* were varied in the following range, from more facilitatory to more depressing: $U:0.05\to 0.9,\;\tau_{rec}:20\to 500\;\text{ms}$ and $\tau_{f}:500\to 20\;\text{ms}$. ***A***, The optimal frequency *r_opt_* as a function of STP properties that gradually and monotonically change the synapse from facilitatory to depressing. *r_opt_* is high for facilitatory synapses and low for depressing synapses. *r_opt_* monotonically decreases as synapses change from facilitatory to depressing. As the basal rate is increased, *r_opt_* decreased for all types of synapses. The circles mark the parameters position for the facilitatory (*s*1, blue) and a depressing (*s*2, red) synapses used as reference in this work. ***B***, *G_max_* as a function of STP properties that gradually and monotonically change the synapse from facilitatory to depressing. Similar to *r_opt_*, *G_max_* also decays rapidly for more depressing types and for higher basal rates. ***C***, Relationship between *G_max_* and *r_opt_*. Notice the approximately linear relationship for facilitatory synapses, with the slope steadily decreasing with increasing *r_bas_*. This summarizes our prediction that higher basal firing rates will constrain the amplitudes of activity distribution-dependent gains and optimal encoding rates. ***D***, Optimal distribution of presynaptic activity that maximizes the gain *G*. The change from sparse (OD∼0) to dense (OD∼1) optimal distribution is abrupt and occurs approximately at the same STP region for different stimulus durations (*T_s_*). However, the transition point where *OD* changes from sparse to dense code is strongly modulated by *r_bas_*, higher basal rates allow for sparse code only for much more facilitatory synapses. ***E***, Optimal distribution of rate as a function of the three key model parameters (*U*, *τ_rec_*, and *τ_f_*). The variable *U* is the most influential in defining the optimal encoding distribution, with U∼0.45 defining the *OD* transition point for $T_{s}=40\;\text{ms}$ and $r_{bas}=0.5\;\text{Hz}$. Marker sizes represent *OD* values, with large ones for *OD *=* *1 and small ones for OD∼0. Effects of STP attributes on maximum gain of the neural population can be seen in Extended Data Figure 3-1.

We define *R_ext_* as a fraction of *R_bas_* to keep the same signal-to-noise ratio ($R_{ext}/R_{bas}$) for populations of different sizes *N*. We find that *r_opt_* is fixed given the STP parameters and *r_bas_* (see Results), therefore by defining *r_δ_* ($R_{ext}/N$) as a fraction of *r_bas_* ($R_{bas}/N$) we reach the interesting consequence of *OD* being independent of any particular choices of population size (Eq. 7). That is, given the same STP parameters and value of *r_bas_*, populations of different sizes will optimally encode the same stimulus intensity (relative to their basal activity) with the same *OD*. Because the optimal encoding rate is constrained by $r_{\delta }<r_{opt}<\infty$, the optimal distribution will also be constrained to 0<OD<1 (see Fig. 3*D*), with values close to zero or one characterizing sparse or dense distributions, respectively.

10.1523/ENEURO.0297-20.2021.f3-1 Extended Data Figure 3-1 Effects of STP attributes on maximum gain of the neural population. ***A***, Similar to Figure 3 but with $T_{s}=100ms$. ***B***, Similar to Figure 3 but with $T_{s}=300ms$. Both panels reproduce the main findings of Figure 3, showing that the distribution-dependent gain is high for facilitatory synapses and it is strongly affected by the basal rate even if we consider longer integration time windows. The relative importance of the recovery time constant and facilitation time constant in defining the optimal distribution *OD* grow with larger *T_s_* (large circles for *OD *=* *1 and small circles for OD∼0), but the facilitation factor *U* keeps being the most relevant attribute in defining the *OD*. Download Figure 3-1, EPS file.

### Optimal rate ($r_{\text{opt}}$) and maximum gain ($G_{\text{max}}$) estimation

Equation 6 describes the gain *G* obtained by encoding a stimulus *R_ext_* into *N_ext_* units (with rates increased by $r_{ext}=R_{ext}/N_{ext}$) as opposed to *N* units (with rates increased by $r_{\delta }=R_{ext}/N$). The peak of this function (*G_max_*) is achieved by an optimal number of encoding units *N_ext_* = *N_opt_* with their rate increased by $r_{opt}=R_{ext}/N_{opt}$. This maximum point can be found by taking the derivative of the gain function with respect to *r_ext_* and setting it equal to zero,

(8) $$\frac{dG}{dr_{ext}}=\frac{d}{dr_{ext}}(\frac{(N-N_{ext})\cdot Q_{bas}^{s}+N_{ext}\cdot Q_{ext}^{s}-N\cdot Q_{bas}^{s}}{N\cdot Q_{\delta }^{s}-N\cdot Q_{bas}^{s}}-1)=0,$$

given that $\frac{dQ_{\delta }}{dr_{ext}}=0$, this can be further simplified into

(9) $$\begin{array}{l} \frac{Q_{ext}^{s}-Q_{bas}^{s}}{Q_{ext}^{s\prime }}-r_{ext}=0, \end{array}$$

where $Q_{ext}^{s\prime }=dQ_{ext}^{s}/dr_{ext}$ and the value of *r_opt_* is the solution of the equation 9. This solution is independent of the stimulus intensity *R_ext_* and population size *N* (see results in Fig. 2*F*).

For the optimal rate *r_opt_*, the gain (Eq. 6) can be written as

(10) $$G_{max}=100\cdot (\frac{r_{\delta }\cdot (Q_{opt}^{s}-Q_{bas}^{s})}{r_{opt}\cdot (Q_{\delta }^{s}-Q_{bas}^{s})}-1).$$

Assuming that $Q_{\delta }^{s}$ is linear with slope *S^s^* for small *r_δ_*, that is, $Q_{\delta }^{s}=Q_{bas}^{s}+S^{s}\cdot r_{\delta }$ (see below, Linear approximation of $Q_{\delta }^{s}$), then *G_max_* can be further simplified into

(11) $$G_{max}=100\cdot (\frac{Q_{opt}^{s}-Q_{bas}^{s}}{r_{opt}\cdot S^{s}}-1),$$

which makes *G_max_* independent of the stimulus intensity *R_ext_* and population size *N*.

### Combined optimal rate ($r_{\text{opt}}^{\text{com}}$) and maximum gain ($G_{\text{max}}^{\text{com}}$) estimation

When an axon branches to connect to different targets, STP properties might be target dependent. In the case of excitatory fibers driving feedforward excitation-inhibition (FF-EI) motifs, with synapses type 1 (*s*1) directly exciting a readout neuron and synapses type 2 (*s*2) driving the local inhibitory circuit (Fig. 1*C*), the combined gain is given by

(12) $$\begin{array}{ll} \frac{G^{com}}{100}= & \frac{r_{\delta }}{r_{ext}}\cdot (\frac{Q_{ext}^{s1}-Q_{bas}^{s1}}{Q_{\delta }^{s1}-Q_{bas}^{s1}}-\frac{Q_{ext}^{s2}-Q_{bas}^{s2}}{Q_{\delta }^{s2}-Q_{bas}^{s2}}), \end{array}$$

To find the activity distribution that maximizes the combined gain, we take the derivative of *G^com^* with respect to *r_ext_*, set it equal to zero and, assuming again that $Q_{\delta }^{s}$ is linear with slope *S^s^* for both synapses, find the equivalence

(13) $$\begin{array}{ll} \frac{Q_{bas}^{s1}-Q_{ext}^{s1}+r_{ext}\cdot Q_{ext}^{s1\prime }}{S^{s1}} & =\frac{Q_{bas}^{s2}-Q_{ext}^{s2}+r_{ext}\cdot Q_{ext}^{s2\prime }}{S^{s2}}, \end{array}$$

for which the solution, $r_{ext}=r_{opt}^{com}$, is independent of the stimulus intensity *R_ext_* and population size *N*. The optimal combined gain is then

(14) $$\begin{array}{ll} G_{opt}^{com}= & 100\cdot (\frac{Q_{opt}^{s1}-Q_{bas}^{s1}}{r_{opt}^{com}\cdot S^{s1}}-\frac{Q_{opt}^{s2}-Q_{bas}^{s2}}{r_{opt}^{com}\cdot S^{s2}}), \end{array}$$

which is also independent of the stimulus intensity *R_ext_* and population size *N*.

### Numerical simulations

As a proof of concept of the potential relevance that the estimated presynaptic gains could have on postsynaptic targets, we performed numerical simulations of a conductance-based integrate-and-fire (I&F) neuron model acting as the readout device for a FF-EI circuit (See section Sparse code identification by a postsynaptic neuron mode). The I&F model’s membrane voltage *V_m_* is described by

(15) $$\begin{array}{l} C_{m}\frac{dV_{m}}{dt}=g_{e}(V_{e}-V_{m})+g_{i}(V_{i}-V_{m}) \end{array},$$

where *C_m_* = 250 pF is the membrane capacitance, *g_e_* and *g_i_* are, respectively, the excitatory and inhibitory input conductances and *V _e_* = 0 mV and *V _i_* = –75 mV are the excitatory and inhibitory synaptic reverse potentials. When a spike occurs, the membrane voltage is reset to *V _reset_* = –60 mV and held at this value for a refractory period of 2 ms. The synapses were modeled by *α*-functions (Kuhn et al., 2004) with time constants *τ_e_* = 0.5 ms for excitatory and *τ_i_* = 2 ms for inhibitory synapses.

The presynaptic population consisted of *N *=* *160,000 units that connected to the I&F neuron in a FF-EI arrangement. The population stationary basal rate was *R_bas_* = 80 kHz, with the individual basal rate *r_bas_* = 0.5 Hz. At the stationary basal rate, the synaptic states are described by

(16) $$\begin{array}{l} u_{bas}=U\frac{1+\tau_{f}r_{bas}}{1+U\tau_{f}r_{bas}}\\ x_{bas}=\frac{1}{1+u_{bas}\tau_{rec}r_{bas}}\\ PRR_{bas}^{s}=u_{bas}x_{bas}r_{bas} \end{array},$$

where $PRR_{bas}^{s}$ is the expected rate of *PRR* by each synapse with STP parameters $\left\{U,\tau_{rec},\tau_{f}\right\}$.

We simulate a neuron that, during stationary basal activity, is kept in the fluctuation-driven regime through excitation-inhibition input balance (Kuhn et al., 2004). While excitation is provided directly by *s*1, disynaptic inhibition is modulated by *s*2 in a linear fashion,

(17) $$\begin{array}{l} \lambda_{i}=aPRR^{s2}. \end{array}$$

The inhibitory firing rate that keeps the target neuron membrane potential fluctuating around the mean value of $\bar{V_{m}}$ during stationary basal activity can be approximated by a linear function of the excitation (adapted from Kuhn et al., 2004):

(18) $$\begin{array}{l} \lambda_{i}\approx -\frac{(V_{e}-\bar{V_{m}})B_{e}\tau_{e}}{(V_{i}-\bar{V_{m}})B_{i}\tau_{i}}PRR_{bas}^{s1}\\ a\approx -\frac{(V_{e}-\bar{V_{m}})B_{e}\tau_{e}}{(V_{i}-\bar{V_{m}})B_{i}\tau_{i}}\frac{PRR_{bas}^{s1}}{PRR_{bas}^{s2}} \end{array},$$

where *B_e_* and *B_i_* are the maximum amplitudes for the excitatory and inhibitory synaptic conductances. Equation 18 allows to find the linear scale of Equation 17 that fulfills the condition $\bar{V_{m}}=-53mV$. The inhibitory synapses are kept static (no STP). The extra presynaptic activity happens in blocks of $T_{s}=40\;\text{ms}$ and is defined as sparse (when *N_ext_* = *N_opt_*) or dense (when *N_ext_* = *N*).

### Continuous rate distribution

Although some bursting networks [e.g., cerebellar parallel fibers (PFs)] seem to operate in a quasi-binary fashion (burst or no-burst), it is important to extend the analysis to continuous distributions, which most parts of the brain seem to operate under. We do this by assuming that the distribution of event-related neural firing rates follows a γ distribution, which allows us parameterized control of the sparseness of the neural code (with the mean of the distribution) and of the distribution shape (with the skewness and kurtosis):

(19) $$\begin{array}{l} r_{ext}∼Gamma(k,\theta ), \end{array}$$

where *k* is the shape parameter and *θ* is the scale parameter. When *k *=* *1, this is equivalent to an exponential distribution and, for increasing values of *k*, this becomes a right-skewed distribution, with the skewness approaching zero for higher values of *k* (becoming approximately Gaussian). For each shape parameter, we controlled the mean of the distribution by varying the scale parameter, because for a γ distributed *r_ext_* the expected value is

(20) $$\begin{array}{l} E[r_{ext}]=k\theta . \end{array}$$

For the γ-specified distribution of extra rates and a given presynaptic set of STP parameters, the expected amount of resources released by a population is

(21) $$\begin{array}{l} E[Q_{ext}^{p}]=\int_{r_{\delta }}^{\infty }\frac{Gamma(r_{ext};k,\theta )}{r_{ext}}Q^{s}(r_{ext})dr_{ext}, \end{array}$$

which we solved numerically for two synapse types (*s*1-facilitatory and *s*2-depressing) and a range of rate distributions. The distribution gain *G* for $E[Q_{ext}^{p}]$ was then calculated in relation to the dense case, where *N_ext_* = *N* and $r_{ext}=r_{\delta }$.

A glossary of of key symbols used throughout the work is given in Table 1. All the analyses and simulations were performed in MATLAB and Python. The model simulations were performed using Euler’s method with time step of 0.1 ms implemented in the neural simulator Brian2 (Stimberg et al., 2014). The simulation and analysis code is available on GitHub at https://github.com/luiztauffer/stp-activity-distribution.

## Results

Here, we are interested in a mechanism by which a neuronal network or a single postsynaptic neuron receiving multiple inputs may distinguish between different spiking distributions with the same intensity (e.g., the same number of spikes). This problem is schematically illustrated in Figure 1. Consider two scenarios. In the first scenario, seven spikes arrive from a single presynaptic neuron while others six remain silent (Fig. 1*A*, sparse distribution). In the second scenario, each of the seven presynaptic neurons spikes once. In both trials, the postsynaptic neuron receives seven spikes (Fig. 1*B*, dense distribution). Here, we test the hypothesis that when synapses exhibit STP (facilitation or depression) the two scenarios can be differentiated without any specific tuning of synaptic weights.

Static synapses evoke exactly the same PSC sequence for both sparse and dense distributions (black lines), making them indistinguishable for a readout neuron. However, when synapses are dynamic, short-term facilitation (blue line) enhances the PSC amplitudes compared with the static synapses (compare Fig. 1*A*,*B*, bottom traces). Short-term depression (red line) results in a weaker response as compared with the static synapses (compare Fig. 1*A*,*B*, bottom traces). If the incoming spikes are distributed along different synapses, the sequence of PSCs is identical for all types of synaptic dynamics (compare Fig. 1*A*,*B*, bottom traces).

*In vivo* neural coding is certainly more complex than the above example. However, this simple example suggests that in the case of a neuron receiving synaptic inputs via thousands of noisy synapses, STP could be a mechanism to differentiate between an evoked signal from the background activity fluctuations of the same amplitude, provided the former is encoded as a specific pattern that can exploit the STP properties of the synapses. In the following, we describe how well dynamic synapses could endow feedforward circuits with such activity distribution discrimination properties in low signal-to-noise regimes (Fig. 1*C*).

### Optimal activity distribution with dynamic synapses

We implemented dynamic synapses with the rate-based TM model (Tsodyks et al., 1998; Eq. 2). In this model, the instantaneous *PRR* depends on the resource release probability (*u*^+^) and the proportion of available resources (*x*^–^), which have their dynamics guided by the choice of STP model parameters $\left\{U,\tau_{f},\tau_{rec}\right\}$. For a transient increase in firing rate, a facilitatory synapse produces an average profile of sustained *PRR*, while a depressing synapse produces an average profile of rapid decaying *PRR* (Fig. 2*A*). Throughout this work, the two reference sets of values for STP types are: *U *=* *0.1, $\tau_{f}=200\;\text{ms}$ and $\tau_{rec}=50\;\text{ms}$ (facilitatory) and *U *=* *0.7, $\tau_{f}=50\;\text{ms}$ and $\tau_{rec}=200\;\text{ms}$ (depressing).

To quantify the effects that different profiles will have on the presynaptic output, for varying transient increases in firing rate (*r_ext_*), we calculate the total amount of extra resources ($Q_{ext}^{s}$) a synapse releases over a time period of *T_s_* (Eq. 3; Fig. 2*B*). We found that $Q_{ext}^{s}$ varied in a nonlinear fashion as a function of *r_ext_*, with depressing dynamics approaching $Q_{ext}^{s}$ saturation much faster than facilitatory dynamics. The slope of $Q_{ext}^{s}$ (Fig. 2*B*, inset) for depressing synapses is monotonically decreasing, indicating that any increase in the firing rate in those synapses will produce sublinear increase in $Q_{ext}^{s}$, whereas for facilitatory synapses the slope initially grows, indicating that increases in the firing rate of those synapses, up to some point, will produce supralinear increase in $Q_{ext}^{s}$.

In the brain, neurons typically receive inputs from a large ensemble of presynaptic neurons. In the ongoing activity state, these neurons spike at a low-basal firing rate (*r_bas_*) with the total synaptic output of $Q_{bas}^{p}=N\cdot Q_{bas}^{s}$. In the event-related activity state, the firing rate of a subset of presynaptic neurons (*N_ext_*) is transiently increased and the total synaptic output (Eq. 4) changes accordingly. We distribute a fixed event-related population rate increase *R_ext_* into varied numbers of chosen synapses *N_ext_*, each of these chosen synapses increasing its firing rate by *r_ext_*, that is, $R_{ext}=N_{ext}\times r_{ext}$, and report the changes in $Q_{ext}^{p}$.

We found that, for a population of facilitatory synapses, $Q_{ext}^{p}$ varied in a non-monotonic fashion as a function of *N_ext_*, initially increasing up to a peak point, then decreasing (Fig. 2*C*, left). By contrast, for depressing synapses (Fig. 2*C*, right), $Q_{ext}^{p}$ varied in a monotonically increasing fashion. For both facilitatory and depressing synapses, $Q_{ext}^{p}$ converged to their respective $Q_{\delta }^{p}$ when the total extra input rate *R_ext_* was distributed over all the neurons such that *N_ext_* = *N* and $r_{ext}=r_{\delta }=R_{ext}/N$.

These results suggest that, when synapses are facilitatory, the total amount of synaptic resources released during a event-related activity state is maximized when event-related spiking activity is confined to a small number of synapses. $Q_{ext}^{p}$ was smaller than $Q_{\delta }^{p}$ when *R_ext_* was distributed into a small subset of presynaptic neurons, because those chosen neurons spiked at very high rates and the synapses ran out of vesicle resources rapidly. When the event-related input was distributed over all the presynaptic neurons, the $Q_{ext}^{p}$ also decreased because in such a scenario $r_{ext}=r_{\delta }=R_{ext}/N$ was too small to fully exploit the benefits of synaptic facilitation. In contrast to the facilitatory synapses, for depressing synapses it was more beneficial to distribute the event-related spiking activity over the whole input ensemble to maximize the total amount of synaptic resources released. In this condition, $r_{ext}=r_{\delta }$ was small enough to avoid any losses in vesicle release caused by depression.

### Activity distribution-dependent gain

To further quantify the effect of distribution of event-related activity over the input ensemble (that is, how neurons increase their rate in the event-related phase), we defined the distribution gain *G* as the proportional change in $Q_{ext}^{p}$ in relation to $Q_{\delta }^{p}$ (Eqs. 5, 6). We found that $Q_{\delta }^{p}$ is approximately a linear function of *r_δ_* for a wide range of scenarios (see Materials and Methods) and, because of that, with the dense distribution of the activity (when all the presynaptic neurons change their firing rate by a small amount *r_δ_* in the event-related activity state), even dynamic synapses behave approximately as static synapses. Therefore, *G* can be understood either as a gain over a dense distribution or as a gain over static synapses. For facilitatory synapses, just as for $Q_{ext}^{p}$, *G* follows a non-monotonic curve as a function of *N_ext_*, with a single peak at *N_opt_* (Fig. 2*D*, blue line). By contrast, depressing synapses resulted in negative gains to every distribution, except for *N_ext_* = *N* where *G *=* *0% (Fig. 2*D*, red line).

Next, we estimate *N_opt_* and *G* for a range of extra activity intensities (Fig. 2*E*, for facilitatory synapses). For these calculations, we parameterized the extra activity *R_ext_* as a fraction of the basal firing rate *R_bas_* (correspondingly, *r_δ_* as % of *r_bas_*; see Materials and Methods). We found that, for facilitatory synapses, *N_opt_* increased linearly with the extra activity intensity (Fig. 2*G*), resulting in an optimal encoding rate *r_opt_* which is independent of the input intensity. For depressing synapses, the optimal distribution *N_opt_* = *N* did not change with the extra activity intensity, making the optimal encoding rate always *r_opt_* = *r_δ_*.

Because the presynaptic neurons are assumed to be Poisson processes, an advantage of parametrize *R_ext_* in terms of fraction of *R_bas_* is that it directly translates to signal-to-noise ratio. For the example shown in Figure 2*G*, we found that STP could amplify the presynaptic output for weak signals (which were <10% of the basal activity) by up to 60% if the extra rate was distributed over *N_opt_* synapses as opposed to *N* synapses. For low signal-to-noise ratios ($r_{\delta }<r_{bas}$), the gain at the optimal distribution (*G_max_*) was approximately constant and always positive for facilitatory synapses, while depressing synapses keep *G_max_* = 0 at *N_opt_* = *N* (Fig. 2*G*). Finally, we analytically show that the independence of *r_opt_* and *G_max_* from the extra activity intensity is a good approximation for a wide range of basal rates and STP types (see Materials and Methods).

These results suggest that when synapses are facilitatory, the input should be distributed sparsely (or sparse code, that is, only a small set of neurons change their firing rate in the event-related state) to maximize the total amount of synaptic resources released at the downstream neuron. By contrast, when synapses are depressing, the input should be distributed densely (or dense code, that is, all the neurons change their firing rate in the event-related state) to maximize the synaptic resources released at the downstream neuron. Thus, for sparse population activity, while facilitatory synapses are optimally used, depressing synapses are subutilized.

### Effects of STP parameters on optimal rate and gain

Next, we investigated how *N_opt_*, *r_opt_*, and *G_max_* vary with STP parameters. To this end, we systematically changed synapses from facilitatory to depressing by jointly varying the set of parameters: $U=\{0.05,...,0.9\},\;\tau_{rec}=\{0.02,0.5\}$ ms and $\tau_{f}=\{0.5,...,0.02\}$ ms. We found that *r_opt_* decayed exponentially as the synapses became more depressing (Fig. 3*A*). This follows from the fact that facilitatory synapses profit from high firing rates and depressing synapses avoid negative gains at lower rates.

The maximum gain *G_max_* also decreased exponentially as synapses were systematically changed from facilitatory to depressing (Fig. 3*B*). We found that the relationship between gain and optimal rate was linear from mildly to strongly facilitatory synapses (Fig. 3*C*), with larger basal rates constraining the optimal conditions to lower rates with lower gains.

Interestingly, increasing the basal firing rate *r_bas_* substantially reduced *r_opt_* and *G_max_*. This is surprising because, at such low values of spiking rates, STP effects are hardly perceivable in traditional paired-pulse ratio analyses. The high value of *G_max_*, when the system operates at low *r_bas_*, happens because of synapses taking advantage of the nonlinearities in their individual $Q_{ext}^{s}$ (Fig. 2*B*). Increased basal activity attenuates these nonlinearities, therefore impairing the distribution-dependent gain.

### Relationship between facilitatory synapses and sparse coding

We quantified the optimal distribution of an evoked neural signal by *OD* (see Materials and Methods). High *OD* (OD→1) indicates a dense distribution in which many neurons spike to encode the extra activity, whereas low *OD* (OD→0) indicates a sparse distribution. We found that *OD* changed abruptly from sparse to dense as synapses were changed from facilitatory to depressing (Fig. 3*D*). Facilitatory synapses yielded maximum response for sparse while depressing synapses yielded maximum response (avoid negative gains) for dense distributions. The transition point from sparse to dense *OD* did not depend on the stimulus duration. However, the basal rate strongly modified the transition point, with higher *r_bas_* allowing only strongly facilitatory synapses to take advantage of sparse distributions. This configuration remained independent of the stimulus intensity as long as the circuit operates at low signal-to-noise conditions ($r_{\delta }/r_{bas}<1$; Fig. 2*G*).

In the above, we changed the synapses from facilitatory to depressing by linearly modifying the whole set of parameters together. Next, we systematically varied each of the STP parameters independently and measured the *OD* for maximum gain. We found that the transition region was primarily governed by the facilitation factor *U* (U ∼ 0.45), with a weak dependence on *τ_rec_* and *τ_f_* (Fig. 3*E*). The relative contribution of *τ_rec_* and *τ_f_* became more relevant at higher *T_s_* (Extended Data Fig. 3-1).

These results clearly highlight the importance of the stationary basal rate in how well the synaptic gain modulation operates, as only low *r_bas_* allows for significant gains. Importantly, the switch-like behavior of the optimal distribution indicates that, for a given population code, there is a robust range of STP attributes that could produce positive gains. This transition point seems to be relatively independent of the signal duration but is strongly affected by *r_bas_*. Finally, having a low initial release probability (defined in the model by a low *U*) seems to be the preeminent feature in defining the optimal *OD*.

The Equation 7 suggests that OD is independent of the population size (*N*). However, there is a lower limit of *N* below which the sparsity argument does not hold. We have shown that given the STP parameters, there is an optimum firing rate *r_opt_* at which signal carrying neurons should operate to maximize the gain (Fig. 2*F*). For a given rate it is optimal to distribute spikes over *N_opt_* input channels (Fig. 2*F*). However, when $N=N_{opt}$, then clearly the optimal distribution will not be sparse. The argument for sparseness arises when $N>>N_{opt}$. When $N>>N_{opt}$, and we increase *N* while keeping all other parameters constant, *OD* will decrease. However, if we change *N* while keeping all other parameters constant, the signal-to-noise ratio will change. The signal-to-noise ratio is defined as $R_{ext}/R_{bas}$, where $R_{bas}=N\times r_{bas}$ and if we change *N*, *R_bas_* will also change. In order to maintain the signal-to-noise ratios comparable for low and high *N* scenarios, we need to scale *R_ext_* accordingly. Therefore, here, we defined *R_ext_* in proportion to *R_bas_* so that it can accommodate the changes in *N*. With this choice, *OD* is indeed independent of *N* (see Eq. 7).

### Effects of different sources of enhancement on *G_max_*

The enhancement of the output at facilitatory synapses could, in principle, have many causes (Valera et al., 2012; Thanawala and Regehr, 2013; Jackman and Regehr, 2017). Using the TM model (Eq. 1), we phenomenologically accounted for two important sources: a low initial release probability which sequentially increases with each incoming spike (Jackman et al., 2016) and fast replenishment of readily available resources (Crowley et al., 2007). The first characteristic is mimicked by a low facilitation factor *U*, which determines the initial release probability after a long quiescent period and the proportional increase in it after each spike. The second mechanism is captured by a fast recovery time constant *τ_rec_*.

**Figure 4.**
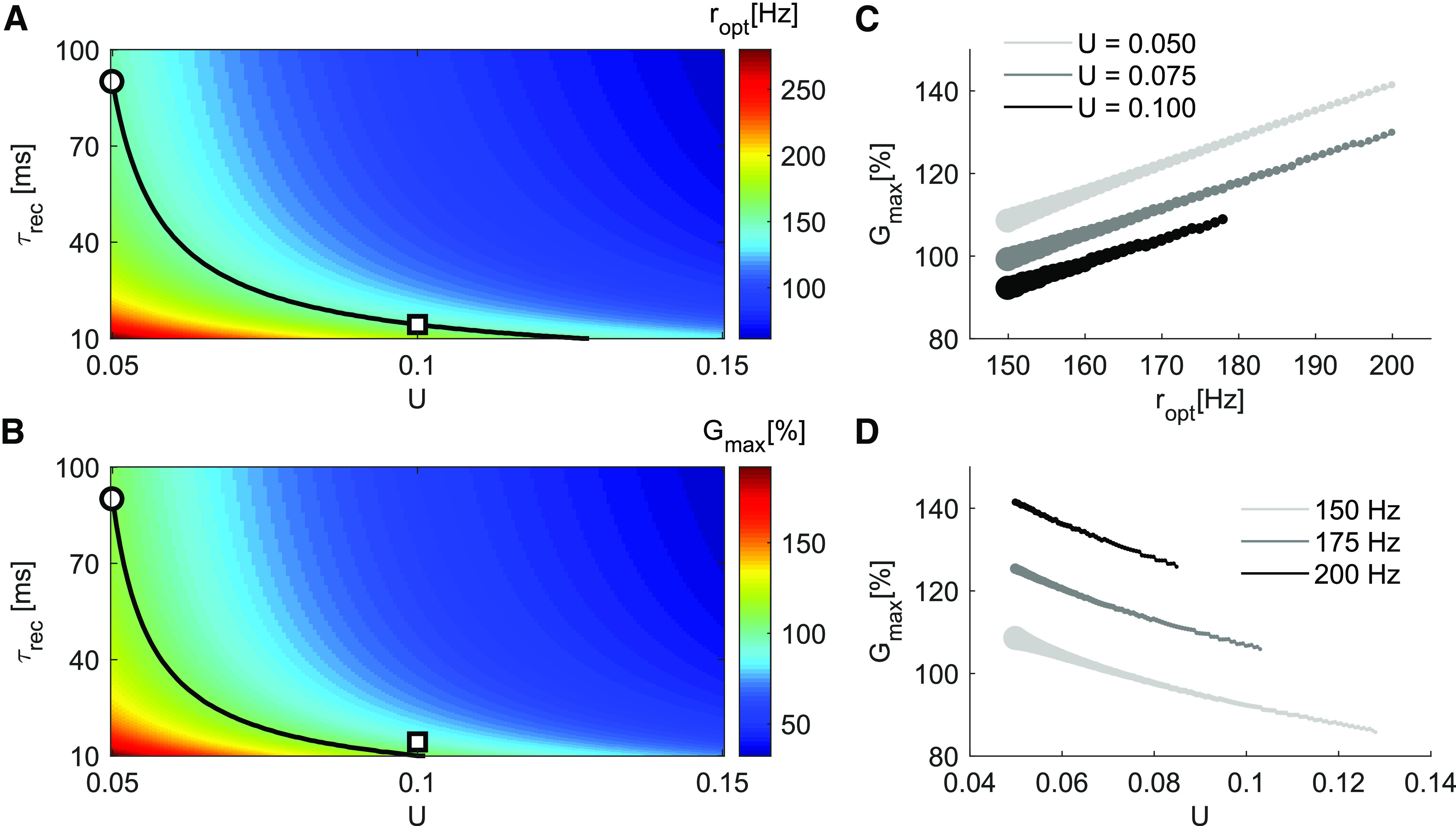
Effects of resources recovery time constant *τ_rec_* and facilitation factor *U* on *G_max_* and *r_opt_* for $T_{s}=40\;\text{ms}$, for facilitatory synapses. ***A***, The *r_opt_* surface, as a function of *U* and *τ_rec_*, shows that a given optimal encoding rate can be matched by different combinations of synaptic parameters. For example, the iso-frequency curve of 150 Hz (black line) can be achieved with either $\left\{U=0.05,\;\tau_{rec}=90\;\text{ms}\right\}$ (°) or $\left\{U=0.1,\;\tau_{rec}=15\;\text{ms}\right\}$ (□). ***B***, The *G_max_* surface, as a function of *U* and *τ_rec_*, shows that the same maximum gain can observed for many different combinations of *U* and *τ_rec_*. The black line shows the contour for $G_{max}=109\%$. The two configurations with same *r_opt_* marked in panel A have distinct gains (°=109%, □=92%). ***C***, We fix *U* and vary *τ_rec_* (circle sizes) to match *r_opt_* (*x*-axis), then observe the gain. Larger values of *U* systematically produce smaller gains. Recovery time has a lower boundary $\tau_{rec}=10\;\text{ms}$. ***D***, *G_max_* as a function of *U* for three different values of *r_opt_*. Larger values of *U* require smaller values of *τ_rec_* (circle sizes) to match the same *r_opt_*, but as a consequence the gain decreases as we increase *U*. Effects of resources recovery time constant *τ_rec_* and facilitation factor *U* on *G_max_* and *r_opt_* for facilitatory synapses can be seen in Extended Data Figure 4-1.

We systematically varied *U* and *τ_rec_* and measured *G_max_* and *r_opt_*. We found that several different combinations of *U* and *τ_rec_* resulted in the same optimal distribution gain and rate. However, when we changed *U* and *τ_rec_* while keeping the *r_opt_* fixed, *G_max_* could no longer be kept constant and vice versa. For instance, the two parameter sets $\left\{U=0.05,\tau_{rec}=90\;\text{ms}\right\}$ and $\left\{U=0.1,\tau_{rec}=15\;\text{ms}\right\}$ gave $r_{opt}=150Hz$ (Fig. 4*A*), but the first parameter set gave $G_{max}=109\%$ and the second parameter set gave *G_max_* = 92% (Fig. 4*B*). Holding *U* fixed and choosing *τ_rec_* to match with different *r_opt_* showed that *G_max_* consistently dropped for higher *U* (Fig. 4*C*,*D*).

10.1523/ENEURO.0297-20.2021.f4-1 Extended Data Figure 4-1 Effects of resources recovery time constant *τ_rec_* and facilitation factor *U* on *G_max_* and *r_opt_* for facilitatory synapses. ***A***, Similar to Figure 4 but with *T_s_* = 100 ms. ***B***, Similar to Figure 4 but with *T_s_* = 300 ms. Both panels reproduce the main findings of Figure 4, showing that a given optimal encoding rate can be matched by different combinations of synaptic parameters, but resulting in different gains. Download Figure 4-1, EPS file.

These results indicate that, in terms of maximum gain *G_max_*, the fine tuning of intracellular mechanisms that work to steadily increase a low initial release probability might be more important than fast vesicle replenishment mechanisms. This remains true for larger *T_s_* (Extended Data Fig. 4-1).

In summary, our results show that a set of presynaptic STP parameters generates a gain surface *G* that, in principle, could be tuned to match presynaptic population activity characteristics. The optimal rate and the maximum gain are independent of the stimulus intensity for a low signal-to-noise ratio, with facilitatory synapses yielding high gains for sparse distributions while depressing synapses avoid negative gains only with dense distributions. For low basal activity (*r_bas_* = 0.5 Hz) and short duration integration window (*T_s_* = 40 ms) conditions, the parameter *U* is the principal determinant of the optimal distribution. Furthermore, lower *U* yields a higher gains than lower *τ_rec_* when the optimal encoding rate is kept constant.

### Feedforward inhibition (FFI) and heterogeneous STP

In the above we ignored the fact that presynaptic STP can be target dependent (Markram et al., 1998; Reyes et al., 1998; Rozov et al., 2001; Sun et al., 2005; Pelkey and McBain, 2007; Bao et al., 2010; Blackman et al., 2013; Larsen and Sjöström, 2015; Éltes et al., 2017), and the spike trains coming from the same axon can be modulated by different short-term dynamics at different synapses. In the following, we describe the effects of such heterogeneity in a FF-EI motif (Fig. 1*C*), an ubiquitous circuit motif across the brain (Klyachko and Stevens, 2006; Dean et al., 2009; Isaacson and Scanziani, 2011; Wilson et al., 2012; Jiang et al., 2015; Grangeray-Vilmint et al., 2018).

**Figure 5.**
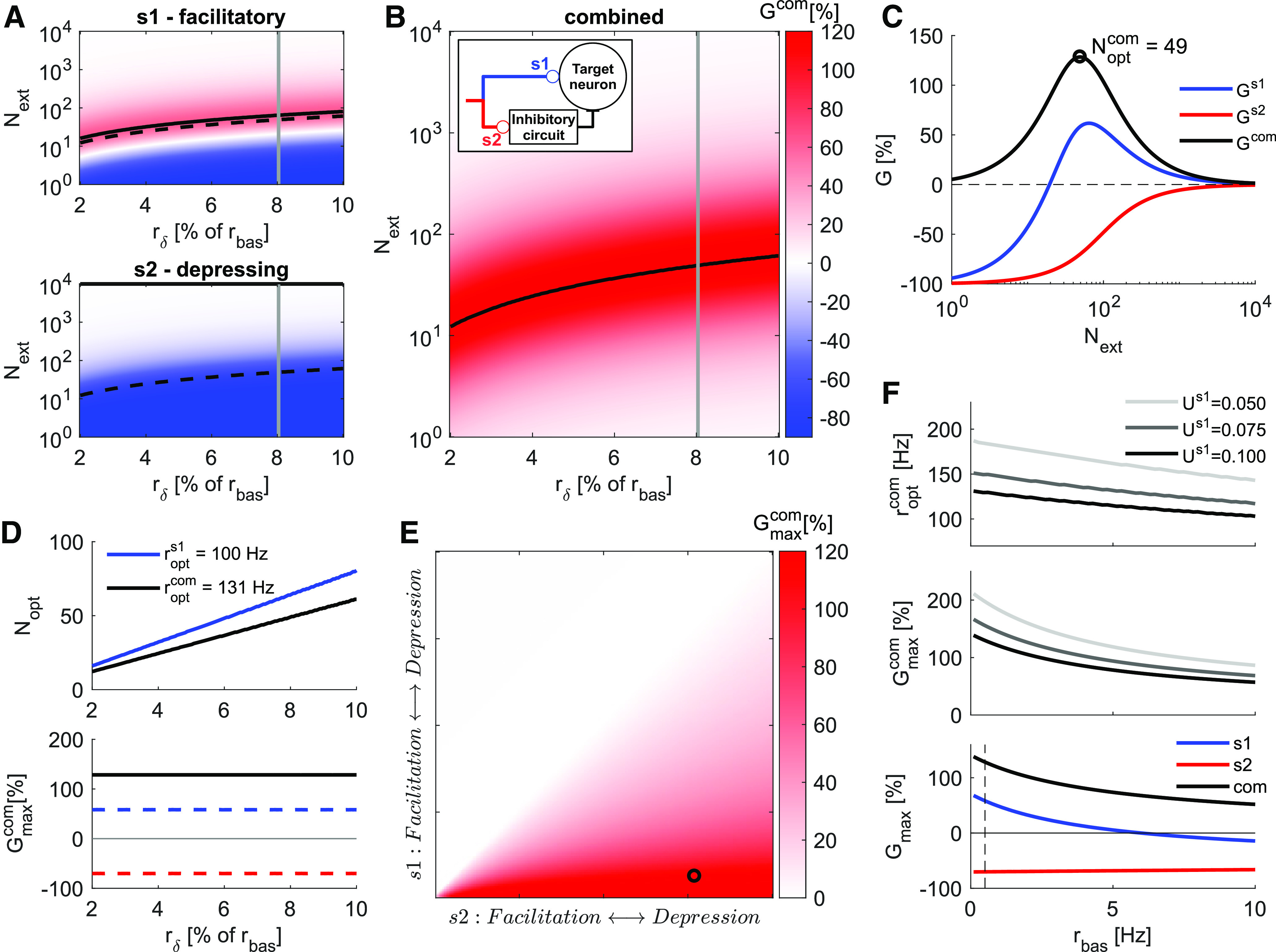
Combined optimal distribution of activity in a FF-EI circuit with target-dependent STP. ***A***, *G* as a function of $r_{\delta }$ and *N_ext_* for a facilitatory synapse (*s*1, top) and for a depressing synapse (*s*2, bottom). This is similar to the Figure 2*E*. ***B***, The combined gain ($G^{com}=G^{s1}-G^{s2}$) of the FF-EI circuit as a function of $r_{\delta }$ and *N_ext_* obtained by combining the gains of the FFE and FFI branches. The black line marks the $N_{opt}^{com}$ for every stimulus intensity $r_{\delta }$ and is represented with dashed black lines in panel ***A***. In-box, Schematic of the FF-EI circuit. ***C***, Gain as a function of *N_ext_* for $r_{\delta }=8\%$ of *r_bas_* (gray lines in panels ***A***, ***B***). *G^com^* inherits the non-monotonicity from $G^{s1}$ (blue, compare with Fig. 2*D*). The gain for a depressing synapse is negative ($G^{s2}$, red) for every $N_{ext}<N$. ***D***, *Nopt* as a function of $r_{\delta }$ produces iso-frequency lines (compare with Fig. 2*F*). $r_{opt}^{com}$ is markedly larger than $r_{opt}^{s1}$ (top). $G_{max}^{com}$ is independent of $r_{\delta }$ (for $r_{\delta }<r_{bas}$, compare with Fig. 2*G*). Gain for both synapse types at $r_{opt}^{com}$ (dashed lines). The small decrease in synaptic gain for *s*1 is compensated by putting *s*2 in a very negative gain region (bottom). ***E***, $G_{max}^{com}$ surface for different combinations of STP characteristics of *s*1 and *s*2. Notice that $G_{max}^{com}$ steadily increases for s1→Fac or s2→Dep, and that $G_{max}^{com}=0$ whenever *s*1 is more depressing than *s*2. The circle marks the specific {s1,s2} combination used in the other panels and throughout the work. ***F***, Effects of ongoing basal activity *r_bas_* on optimal conditions for $T_{s}=40\;\text{ms}$. Increasing basal activity decreases the combined optimal rate (top) and combined maximum gain (center). Results for three different *U* at the facilitatory synapse. Increasing basal activity consistently decreases *G_max_*. $G_{max}^{com}$ decay happens mostly because of decay of the positive gain at the facilitatory synapse *s*1 (blue), while the negative gain at the depressing synapse *s*2 is kept negative and change only slightly (red). Dashed vertical line marks the basal activity used for most part of our analysis, $r_{bas}=0.5\;\text{Hz}$, where both branches contribute significantly to increase the combined gain (bottom).

We extend our previous analysis to a scenario in which the presynaptic population makes synaptic contacts not only with a readout neuron, but also with the local inhibitory population which projects to the readout neuron creating the FF-EI motif. Both, the readout neuron and the inhibitory group receive the same spike trains via two different types of synapses, *s*1 and *s*2 (Fig. 1*C*). Because the presynaptic population activity is the same for both synapses ($r_{bas}=0.5\;\text{ms},\;T_{s}=40\;\text{ms}$), the differences in gain (*G*) are governed by the STP properties of the two synapses. Figure 5*A* shows *G* for a facilitatory (*s*1, *U *=* *0.1, $\tau_{f}=200\;\text{ms},\;\tau_{rec}=50\;\text{ms}$) and a depressing (*s*2, *U *=* *0.7, $\tau_{f}=50\;\text{ms},\;\tau_{rec}=200\;\text{ms}$) synapse.

In the case of a FF-EI network, those two synapse types may be associated with the two branches, for example *s*1 to the feedforward excitation (FFE) branch (targeting a principal neuron) and *s*2 to the feedforward inhibition (FFI) branch (targeting local interneurons which eventually project to principal neurons; Fig. 5*B*, inset). In this arrangement, the combined gain is determined by the two branches $G^{com}=G^{s1}-G^{s2}$. We found that the combined gain of the FF-EI circuit also varied non-monotonically as a function of *N_ext_* and peaked at $N_{opt}^{com}$ which corresponded to the combined optimal encoding rate $r_{opt}^{com}$ (Fig. 5*B*,*C*). Note that the combined maximum gain of the FF-EI circuit is larger than the gain obtained via the FFE branch with facilitatory synapses alone (Fig. 2*C*). This substantial increase is a consequence of the strictly negative profile of $G^{s2}$. When the extra input is distributed in $N_{opt}^{com}$ units (sparse coding), the depressing branch of the FF-EI drove the local inhibitory group with weaker strength than a scenario in which $N_{ext}=N$ (dense coding). Therefore, with sparse distribution of the input, the readout neuron experienced stronger excitation from the FFE branch and weaker inhibition from FFI branch.

Similar to the behavior of facilitatory synapses, in the FF-EI network $N_{opt}^{com}$ increased linearly as a function of $r_{\delta }$, maintaining a constant optimal encoding rate $r_{opt}^{comb}$ (Fig. 2*D*, top). We also observed that $r_{opt}^{comb}$ was larger than $r_{opt}^{s1}$ ($N_{opt}^{com}<N_{opt}^{s1}$), making the isolated gain of *s*1 suboptimal. However, this can be compensated by putting *s*2 into a very negative gain region (Fig. 5*D*, bottom, red dashed line), with a sparse distribution of the inputs. We show analytically that $r_{opt}^{com}$ and $G_{max}^{com}$ are independent of the extra rate for a wide range of conditions (see Materials and Methods).

We extended this analysis to a large range of {s1,s2} STP combinations by gradually changing the set of parameters $\left\{U,\tau_{f},\tau_{rec}\right\}$ (Fig. 5*E*). We found that $G_{max}^{com}$ increased monotonically when we made the synapse *s*1 more facilitatory or when we made the synapse *s*2 more depressing. The anti-diagonal (where s1=s2) marked the region of zero gain and any point above it (*s*2 more facilitatory than *s*1) resulted in $G_{max}^{com}=0$, whereas any point below it (*s*1 more facilitatory than *s*2) resulted in $G_{max}^{com}>0$. As expected, if *s*1 is highly facilitatory and *s*2 highly depressing the combined effect will be of very high gains, given that the presynaptic activity is optimally distributed.

### Effects of basal activity on the FF-EI network

Next, we investigated the effects of the stationary basal activity at the combined optimal conditions of a FF-EI network. We found that the optimal rate and optimal gain both decreased as *r_bas_* was increased (Fig. 5*F*). Separation of the individual contributions of *s*1 and *s*2 branches revealed that this decrease was primarily because of a reduction in the gain of facilitatory synapses (*s*1) whereas the strong negative gain of depressing synapses (*s*2) remained approximately unaltered. This suggests that a population of facilitatory synapses will lose most of its activity distribution-dependent gain as the basal firing rate is increased, whereas a population of depressing synapses can preserve this capability even at larger basal rates.

Thus, these results show that a FF-EI network with target-dependent STP can make the discrimination of sparse activity more robust than what could be achieved by the FFE alone. This can be achieved when the excitatory branch is facilitatory while the activation of the inhibitory branch is depressing (by placing *s*1 and *s*2 at the region below the anti-diagonal in Fig. 5*E*).

### Sparse code identification by a postsynaptic neuron model

**Figure 6.**
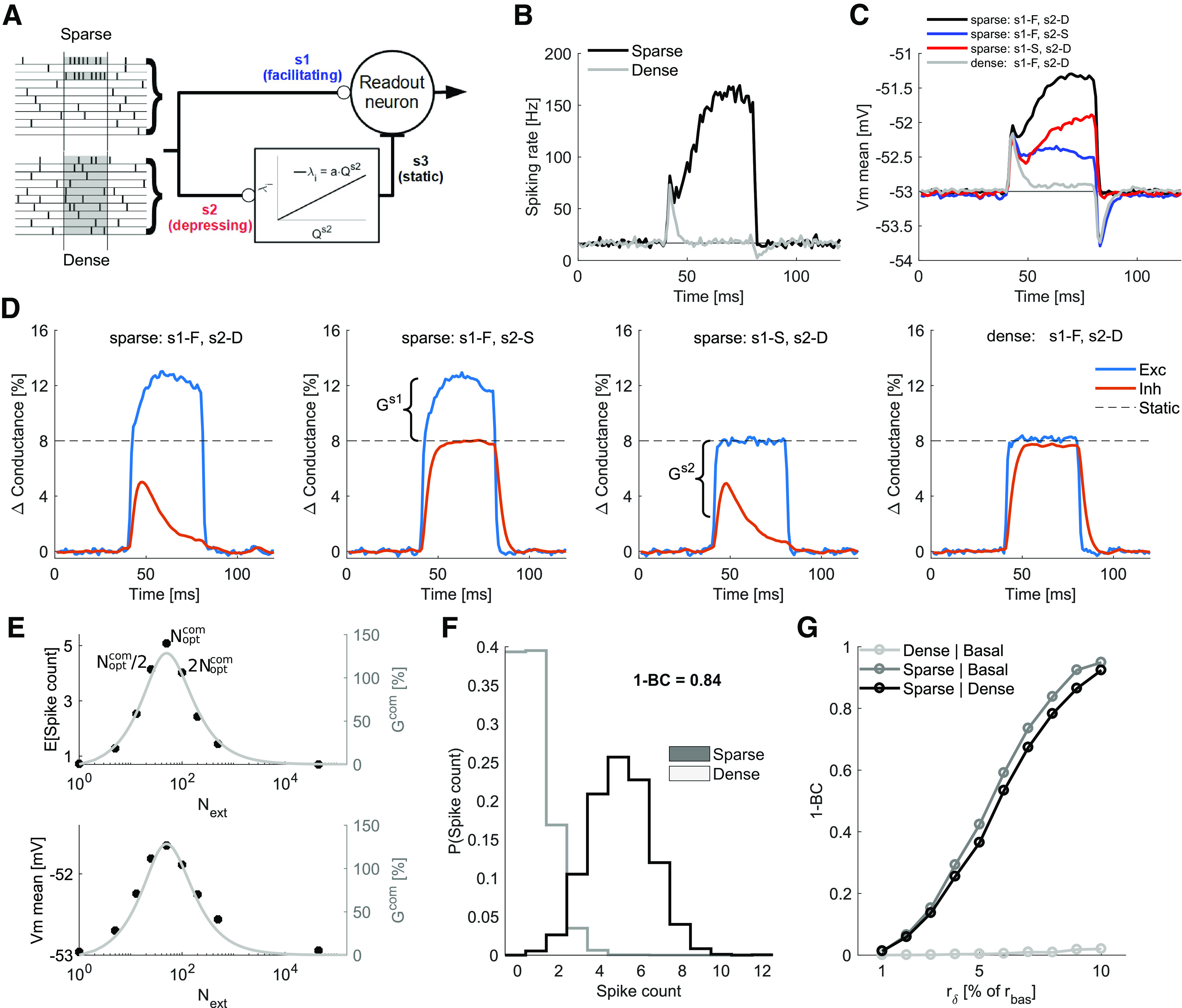
Transfer of STP gain from presynaptic population to postsynaptic neuron. ***A***, Schematic of a readout neuron receiving feedfoward excitation and FFI input. The inhibitory group was driven by the *PRR* of synapses of type *s*_2_ (Eq. 17). Sparse and dense activity patterns are schematically shown. The population temporally ($T_{s}=40\;\text{ms}$ marked in gray) increases its firing rate by $R_{ext}=1...10\%$ of *R_bas_* in two different configurations: sparse (top, *N_ext_* = *N_opt_*) or dense distribution (bottom, $N_{ext}=16,000$). For panels ***B****–****E***, $R_{ext}=8\%$ of *R_bas_*. ***B***, PSTH of the spiking rate (3000 realizations) of readout neuron receiving sparse (black) or dense (gray) distribution of input activity. ***C***, Mean membrane potential for sparse input (black) and dense input (gray) when the synapse *s*_1_ was facilitatory and synapse *s*_2_ was depressing. Red trace, Membrane potential when the synapse *s*_1_ was facilitatory and synapses *s*_2_ was static. Blue trace, Membrane potential when the synapse *s*_1_ was static and synapses *s*_2_ was depressing. The red and blue traces show the contributions from synaptic facilitation (FFE branch) and depression (FFI branch) to the neuron response. ***D***, Changes in the total excitatory and inhibitory conductances for the four configurations of synapses (as show in the panel ***C***). The dashed line marks the conductance changes for the static synapses condition. ***E***, Effect of varying *N_ext_* as a proportion of $N_{opt}^{com}$ on the expected spike count (top, black circles) and the mean membrane potential (bottom, black circles) during the event-related activity period. Both profiles match the combined gain curve (gray line, compare with Fig. 5*C*), with peak at $N_{ext}=N_{opt}^{com}$. ***F***, Probability distribution of output spike counts within *T_s_*. ***G***, Separation (1–BC) between spike count distributions as a function of $r_{\delta }$. The sparse distribution produced increasingly substantial separation when compared with basal (dark gray) and dense distribution (black), whereas the separation was always small when comparing dense distribution with basal activity (light gray).

The ability of STP to amplify the output of a presynaptic population would be functionally relevant only if this amplification is transferred to the postsynaptic side. We tested the postsynaptic effects of the STP based modulation of the presynaptic activity distribution by simulating an I&F neuron model (Eq. 15) as a readout device for a FF-EI circuit (Fig. 6*A*). We simulate a presynaptic population with characteristics similar to the cerebellar molecular layer, a massively feedforward system with properties much alike the ones we have described so far (Ito, 2006).

Specifically, the readout neuron received input from 160,000 presynaptic neurons. The presynaptic background activity was modeled as independent and homogeneous Poisson spike trains with average firing of $r_{bas}=0.5\;\text{Hz}$ ($R_{bas}=80\;\text{kHz}$). In addition, the population of presynaptic neurons increased their firing rate ($R_{ext}=1...10\%$ of *R_bas_*) during a brief time window ($T_{s}=40ms$) to mimic an event-related activity. The extra presynaptic activity was either confined to a small set of presynaptic neurons (*N_ext_* = *N_opt_*, sparse) or distributed over a large number of neurons ($N_{ext}=16,000$, *dense*). The excitatory synapses onto the readout neuron (*s*1) were facilitatory and the STP parameters for each synapse were drawn from a Gaussian distribution (*s*1, $U:mean=.1,s.d.=.02,\;\tau_{rec}:mean=50,s.d.=10\;\text{ms}$ and $\tau_{f}:mean=200,s.d.=40\;\text{ms}$). The FFI activity was modeled as a Poisson process whose firing rate (*λ_i_*; Eq. 9) was linearly dependent on the excitatory input of depressing synapses, whose STP parameters for each synapse were drawn from a Gaussian distribution (*s*2, $U:mean=0.7,s.d.=.14,\;\tau_{rec}:mean=200,s.d.=40\;\text{ms}$, and $\tau_{f}:mean=50,s.d.=10\;\text{ms}$). Maximum weights of each excitatory and inhibitory were drawn from Gaussian distributions (Be:mean=25,s.d.=2.5 nS, Bi:mean=2.,s.d.=.2 nS).

The distribution of the input had a noticeable effect in the output of the target neuron, as shown by the peristimulus time histogram (Fig. 6*B*). While the dense distribution elicited transients at the beginning and ending of the stimulus period because of the inhibition slow time constant, the sparse code elicited a sustained elevated firing rate response throughout the stimulus period. The stimulus induced membrane potential responses for the two types of input patterns (dense and sparse) were also similar to the firing rate responses (Fig. 6*C*). By interchangeably setting *s*1 and *s*2 to static, we identified that both branches contributed significantly to keep the mean membrane potential high in the presence of extra sparse input.

The contribution of each branch becomes clear at the average change in the total excitatory and inhibitory conductances of the readout neuron. When both synapses were dynamic and the stimulus was sparse (Fig. 6*D*, leftmost), the average excitation was larger (because of synaptic facilitation) and the average inhibition was lower (because of synaptic depression) than the average changes caused by a stimulus of the same intensity but with dense distribution (Fig. 6*D*, rightmost). Note how, with dynamic synapses and dense distribution of the stimulus, the conductance changes matched the expected change for static synapses (dashed line). When we kept the stimulus distribution sparse, but interchangeably set *s*1 and *s*2 to static, the conductance trace related to the static branch reached the same value as for the dense distribution and the system was left with the gain produced at the dynamic branch. Dense distributions, therefore, do not exploit the STP nonlinearities and the synapses behave approximately as static, as predicted.

Next, we systematically changed *N_ext_* as percentages of *N_opt_* (*N_ext_* = 1%, 10%, 25%, 50%, 100%, 200%, 400%, 1000% of *N_opt_*, black circles in Fig. 6*E*) and found that both the mean membrane potential and the average spike count during the stimulus period followed profiles that closely matched the predicted *G^com^* curve (Fig. 6*E*). This result confirms that the modulation of the *PRR* from the presynaptic population is faithfully translated into postsynaptic variables (gain estimated at the presynaptic side and membrane potential and spike rate measured on the postsynaptic neuron side). Furthermore, this result also highlights the robustness of this mechanism, even with considerable deviations from the optimal encoding distribution (*N_ext_* = 50% or *N_ext_* = 200% of *N_opt_*, marked as the first black points at left and right from *N_ext_* = *N_opt_*), the evoked responses remained reasonably close to the optimal.

To further assess how individual realizations of the sparse input could be distinguished from a dense input of the same intensity, we sampled the output spike count of the readout neuron for a period of 40 ms during the ongoing basal activity just before the stimulus and during the 40 ms stimulus period for both sparse and dense distributions (Fig. 6*F*). We used the Bhattacharyya coefficient (BC) as a measure of overlap between these sample distributions and 1–BC as a measure of difference (Fig. 6*G*). The dense input had almost complete overlap with the basal condition. On the other hand, the sparse input produced increasingly different response distributions from both the dense input and basal condition, with almost complete separation at $r_{\delta }=10\%$ of *r_bas_*.

Taken together, these results illustrate the potential role of dynamic synapses in amplification of sparse signals at the presynaptic side (*Q^p^*, *G*), even when such signal intensity is just a small fraction of the ongoing basal activity and, therefore, likely to be buried in proportionally large noise fluctuations. In addition, for a dense distribution of the input, the system can preserve short periods (∼10 ms) of increased (decreased) spike probability right after stimulus onset (offset) because of delayed inhibition, which is a known characteristic of FF-EI motifs and might serve as indication of global background rate changes.

### Continuous extra rate distribution

Thus far, we have considered a binary distribution of the extra rate: a fraction of presynaptic cells increased their rate by *r_ext_* or not at all. Although some neural networks might roughly operate in this binary fashion, it is important to ask how would such STP-driven gains operate under continuous distributions, a perhaps more comprehensive way of describing the activity distribution of many neural populations. We therefore estimate the optimal conditions for when the extra presynaptic activity follows a γ distribution (Eq. 19).

**Figure 7.**
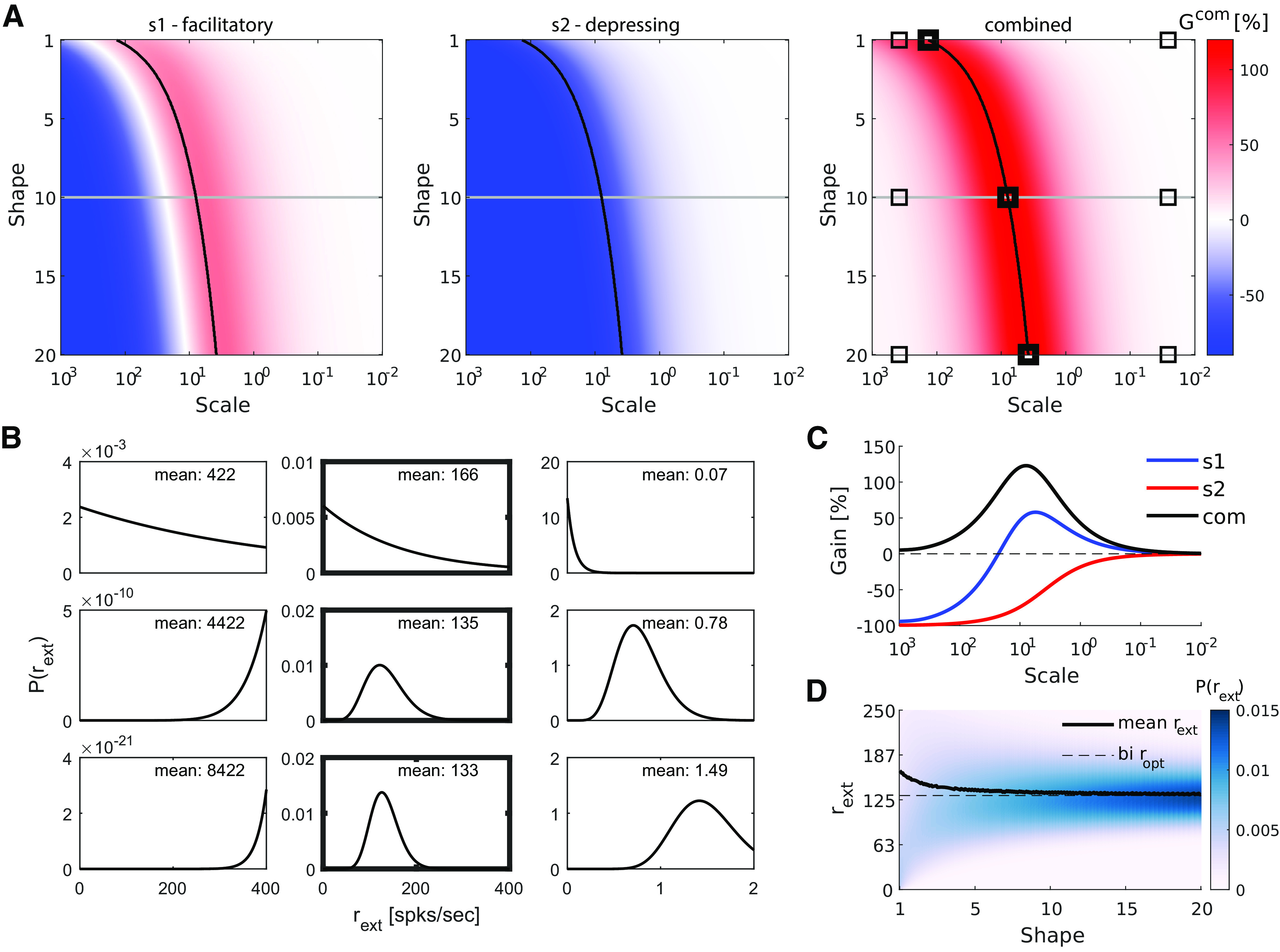
Synaptic gain when firing rates of individual neurons (*r_ext_*) were draw from continuous distributions (γ distribution). ***A***, Gain surfaces for a facilitatory synapse (left), for a depressing synapse (middle) and for the combined effect in a FF-EI (right). Increasing the shape parameter (*k*) moved the distribution from exponential to approximately Gaussian. Decreasing the scale parameter (*θ*) moved the distribution from a high mean and high variance (sparse) to a low mean and low variance one (dense). Black lines mark the *θ* that resulted in maximum combined gain for each value of *k*. Similar to the binary distribution, the $G_{max}^{com}$ is obtained by putting *s*1 in positive and *s*2 in negative gain regions. Note that the gain values are in the same range as in Figure 5*A*,*B*. ***B***, Representative examples of γ functions used to model the distribution *r_ext_*. The nine examples correspond to the points marked in panel ***A***. Changing *k* affects the shape of the distribution: exponential (top row), right-skewed (middle row), and approximately Gaussian (bottom row) shapes. Changing *θ* affects the scale of the distribution (sparsity of the population code): high mean and variance (left column), optimal mean and variance (middle column), and low mean and variance (right column). ***C***, Gain curves for a facilitatory synapse (blue), for a depressing synapse (red), and for the combined effect in a FF-EI (black). These curves were obtained for a fixed *k *=* *10 (gray lines on panel ***A***) and gradually changing *θ*. The gains as a function of the γ activity distribution follow a profile similar to the binary distribution (compare with Fig. 5*C*). ***D***, γ-Distributed *r_ext_* (color plot) as a function of shape parameter. Mean *r_ext_* from the γ distributions obtained with the optimal *θ* for each value of *k* (black lines on panel ***A***). As the γ shape moves from an exponential to a Gaussian one (increasing *k*), the mean of the optimal distribution approaches the *r_opt_* for the binary distribution.

The variation of the shape parameter (1<k<20) changes the distribution from an exponential to a quasi-Gaussian. For each fixed shape, we control the mean (therefore the sparsity; Eq. 20) of the distribution with the scale parameter ($10^{-2}<\theta <10^{3}$). For each set {k,θ} we calculate the expected gain (Eq. 21) yielded by a population of facilitatory synapses (*s*1, 7A left), of depressing synapses (*s*2; Fig. 7*A*, center) and the combined gain (Fig. 7*A*, right). Nine particular parameters choices are demonstrated in Figure 7*B*, where the central panels follow the choices that maximize the combined gain.

We found that, similar to the binary distribution case, the gain for facilitatory synapses followed a non-monotonic curve as a function of *θ* (for a fixed *k*), with negative values at high *θ* (overly sparse distribution), a single peak at the optimal *θ* choice and convergence to 0 at low *θ* (dense distribution). By contrast, depressing synapses showed negative gains, monotonically converging to zero at low *θ*. The combined gain reached high values when *s*1 synapses were in very positive and *s*2 synapses were in very negative operating regions (Fig. 7*C*; see Fig. 7*A*, gray line).

Interestingly, not only the gain magnitudes were very similar to the ones obtained with binary distributions (compare colorbars of Figs. 5*A*,*B* and 7*A*), but also with continuously distributed rates the points of maximum gain were obtained at high mean rates (in relation to *r_δ_*) and, therefore, representative of sparse distributions of the population activity. For increasing values of *k*, the skewness of these distributions approached zero (i.e., became closer to a Gaussian) and the mean *r_ext_* of the optimal *θ* approaches the *r_opt_* obtained by binary distributions. These results further corroborate the effects of the activity distribution-dependent gain modulation in presynaptic populations with STP.

### Continuous basal rate distribution

**Figure 8.**
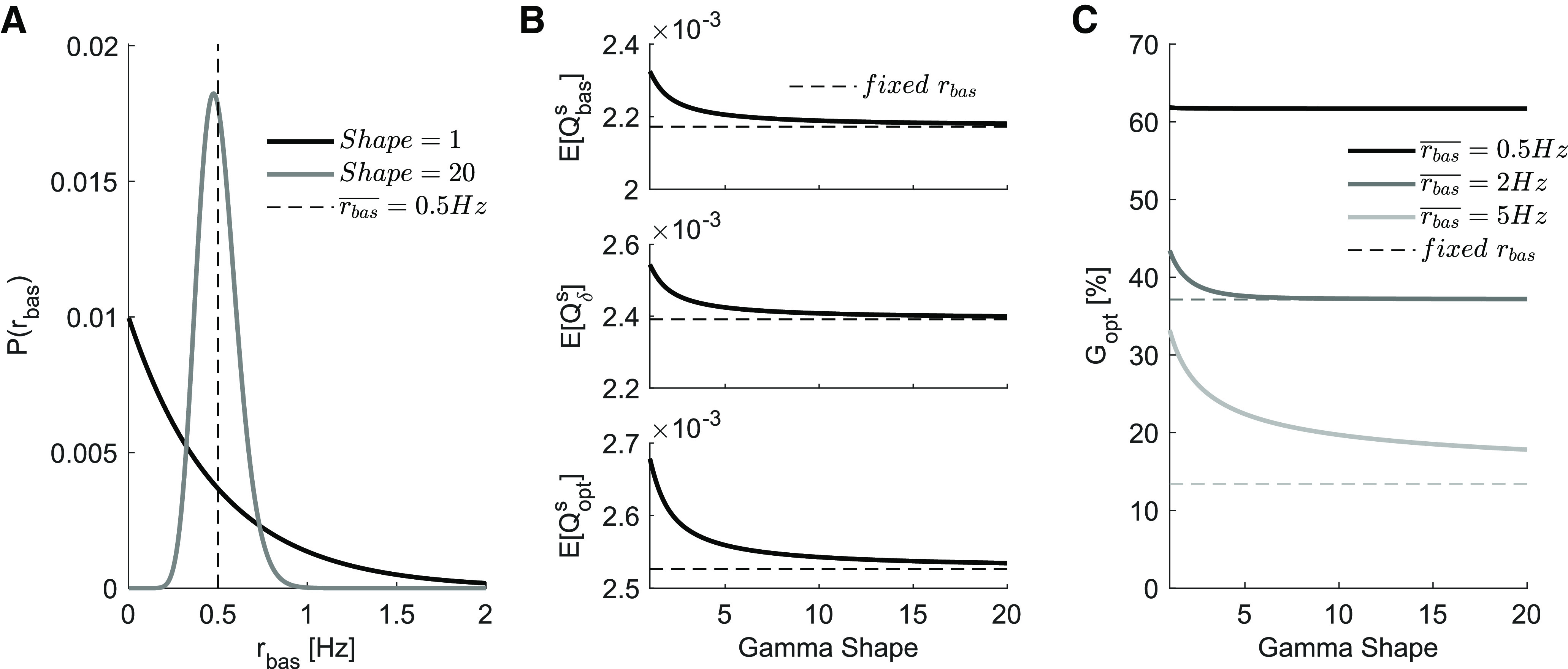
Continuous distribution of *r_bas_*. ***A***, Two configurations of a γ distribution with the same mean value $\bar{r_{bas}}$ = 0.5 Hz. When *k *=* *1, the distribution is exponential and there is a higher probability that any chosen neuron will have *r_bas_* < 0.5 Hz as opposed to *r_bas_* > 0.5 Hz. A higher shape value *k *=* *20 brings the distribution closer to a Gaussian and decreases variance. ***B***, Expected values of $Q_{bas}^{s},\;Q_{\delta }^{s}$ and $Q_{opt}^{s}$ for varied γ shape values. The higher *k* is, the smaller is the variance and the results converge to the estimated values with fixed *r_bas_*. ***C***, Similar to the other variables, the optimal gains start with higher values (when *k* is low) and converge to the estimated values with fixed *r_bas_* for higher *k*. For lower rates the estimates do not diverge much (see overlapping solid and dashed lines when *r_bas_* = 0.5 Hz), but for higher *r_bas_* the increased variance of the continuous distribution has non trivial effects. This indicates that distribution-dependent gains from facilitatory populations might be more resilient to higher basal levels than initially predicted.

In the preceding analysis we assumption that *r_bas_* is fixed and the same for all presynaptic units. A more natural scenario, however, would be to consider a continuous distribution of basal firing rates. We extend our analysis to account for this continuous *r_bas_* scenario in a similar way to what we did for *r_ext_*: we modelled the distribution of basal rates with a γ distribution, with varying shape and scale parameters. The variation of the shape parameter (1<k<20) changed the distribution from an exponential to a quasi-Gaussian (Fig. 8*A*). For each fixed shape, we controlled the mean of the distribution (Eq. 20) with the scale parameter.

We calculated the expected values for $Q_{bas}^{s},\;Q_{\delta }^{s}$, and $Q_{opt}^{s}$ for each distribution shape (Fig. 8*B*) and found that these values converged to the values estimated with a fixed *r_bas_* for higher *k* (quasi-Gaussian) and diverged for lower *k* (exponential). Using these traces, we calculated the gains (Fig. 8*C*) and again found that the results converged to the estimated values for fixed *r_bas_*. For higher *r_bas_*, however, the differences between fixed and continuously distributed *r_bas_* were more pronounced.

The divergence we observed can be explained by the probabilities that any chosen unit will have a rate below or above the mean *r_bas_* value. As discussed above, higher *r_bas_* will hinder the exploitation of STP nonlinearities and, therefore, reduce the possibility of higher gains. For exponential-like distributions, a higher proportion of the population has $r_{bas}<\bar{r_{bas}}$, which reduces this hindering effect, even if a smaller part of the population (for which $r_{bas}>\bar{r_{bas}}$) gets more impaired. As the distribution gets closer to a Gaussian (increase in shape parameter), the proportions of the population with *r_bas_* below or above the $\bar{r_{bas}}$ become almost equal. In the limit of k→∞, the variance of the γ distribution will approximate zero (for our fixed mean) and the gains will converge to the values estimated with fixed *r_bas_*.

It is worth noting that, for higher $\bar{r_{bas}}$, the spike rate variances are also higher and the estimates of gains with fixed *r_bas_* become less accurate. This means that our simplified predictions of the hindering impact that higher *r_bas_* have on the distribution-dependent gains will likely be an overestimate of the actual effects in real neuron populations. In other words, the distribution-dependent gains in facilitatory populations can be more resilient to higher *r_bas_* than what is predicted by a fixed *r_bas_* models.

### From presynaptic gains to postsynaptic rate changes

The readout neuron in our simulations operates in a regime where the presynaptic gains are reliably translated into readout firing rate gains, which is equivalent to saying that the postsynaptic transfer function is independent of the input distribution. However, both synapses and readout neuron dendrites/soma can operate in a nonlinear regime and further transform the presynaptic gain described above. These non-linearities reflect in the transfer function of the neuron, i.e. the probability of an output spike given a certain input.

**Figure 9.**
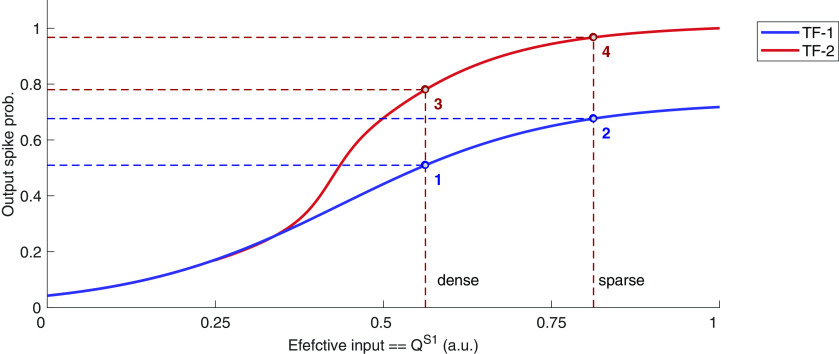
Transfer of presynaptic gains onto postsynaptic rate changes. Distribution-dependent postsynaptic transfer function. For facilitatory synapses, sparse input distributions will yield higher effective input than dense distributions. If both distributions keep the neuron operating under the same transfer function, the presynaptic gain will be efficiently transferred to the postsynaptic rate (1–2 or 3–4). If sparse distributions put the neuron operating under TF-2 while dense distributions keep the neuron under TF-1, the presynaptic gains will be magnified (1–4). Otherwise, if dense distributions put the neuron under TF-2 while sparse distributions keep it under TF-1, the presynaptic gains might be overtaken by the postsynaptic integration effects (2–3).

To identify in which circumstances changes in postsynaptic transfer function may affect the transfer of presynaptic gains into output firing rate, let us consider a neuron with two possible transfer functions (Fig. 9, blue and red curves). The transfer function TF-1 is similar to the one we have considered previously in Figure 6. The TF-2 shows a sharp change. Such a sharp change in the transfer function may arise, for example, because of NMDA receptors. When input is strong, postsynaptic depolarization can remove the Mg^2+^ block and creates a larger EPSP and increase the spike probability (Du et al., 2017). Similarly, nonlinear local dendritic integration (Polsky et al., 2004), input correlations (de la Rocha and Parga, 2005), and voltage dependent ion channels may also create input-dependent changes in the neuron transfer function. When the neuron transfer function can change between TF-1 and TF-2, the output firing rate is not only determined by the effective input (sparse > dense, for *s*1-facilitatory) but also by the qualitative differences in the two transfer functions.

Sparse input distributions will allocate extra incoming spikes as bursts, which could potentially cause extra accumulation of neurotransmitters (for *s*1-facilitatory) in specific dendritic sites, triggering supralinear integration (TF-2, red curve). If a dense input distribution does not attain the triggering of TF-2 and instead keeps operating under TF-1, the difference between presynaptic gains of sparse and dense distributions will be further increased (see the difference between points 1 and 4; Fig. 9).

In cases where both input distributions operate under the same TF the presynaptic gains will be reliably transferred into output rates (compare points 1 and 2 for TF-1 and points 3 and 4 for TF-2 in Fig. 9). Finally, when a dense distribution of inputs makes the output neuron operate under TF-2 and a sparse distribution brings the neuron to operate under TF-1, the presynaptic gains could potentially be overcome (Fig. 9, compare points 2 and 3).

### Linear approximation of $Q_{\delta }^{s}$

**Figure 10.**
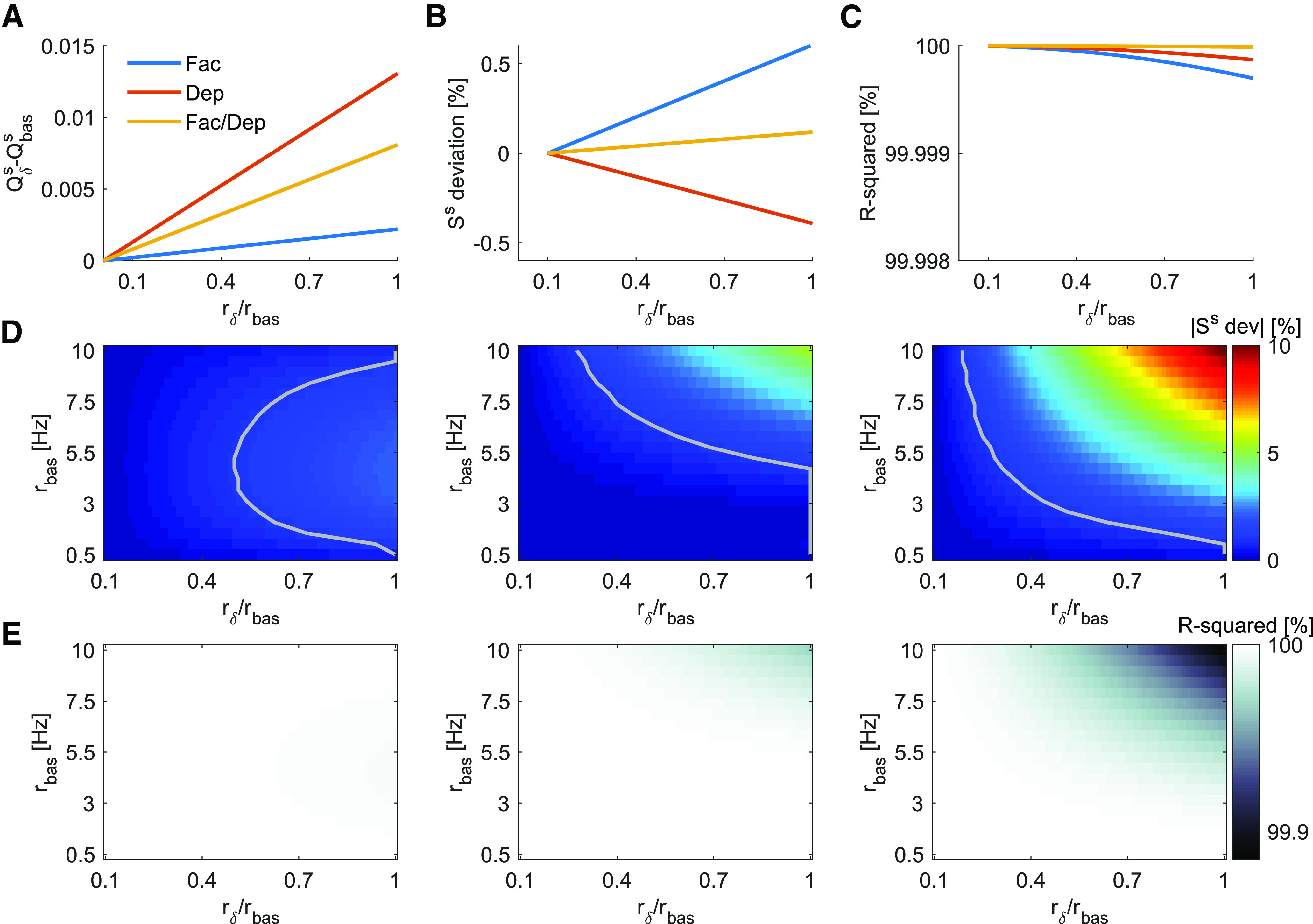
Linear approximation of $Q_{\delta }^{s}$. ***A***, For basal rate $r_{bas}=0.5Hz$ and $T_{s}=40ms,\;Q_{\delta }^{s}$ is approximately linear for all STP regimes, facilitatory (*U *= 0.1,$\tau_{rec}=50ms,\;\tau_{f}=2000ms$), facilitatory/depressing (*U *= 0.4,$\tau_{rec}=100ms,\;\tau_{f}=1000ms$), or depressing (*U* = 0.7,$\tau_{rec}=2000ms,\;\tau_{f}=500ms$). ***B***, Slope deviation for increasing $r_{\delta }$ in comparison to the slope for $r_{\delta }=0.1\cdot r_{bas}$ is always smaller than 0.6% for the three synapse types. ***C***, *R*^2^ is always close to 100% for the three synapse types. ***D***, Absolute value of slope deviation, similar to panel ***B***, but for $r_{\delta }$ departing from several different values of *r_bas_*. The gray line marks $|S^{s}$ dev |=1%. We observe that the linear approximation will work well throughout a large space (left from the gray line) for facilitation (left) and facilitation/depression (middle) regimes and gets a bit more constrained for depressing (right) synapses. ***E***, Similar to panel ***C***, but for $r_{\delta }$ departing from several different values of *r_bas_*.

We solve *Q^s^* numerically (Eq. 3) and show that it behaves linearly for a moderate range of rates in different STP regimes (Fig. 10). The approximation by a linear function, $Q_{\delta }^{s}=Q_{bas}^{s}+S^{s}\cdot r_{\delta }$, allows *G_max_* and *r_opt_* to be independent of the stimulus intensity and population size (Eqs. 13, 14).

To which extent is the linear approximation valid? To investigate this, we solve $Q_{\delta }^{s}$ for gradually increasing $r_{\delta }$ (Fig. 10*A*) departing from a range of different basal levels $r_{bas}=0.5...10$ Hz. We then compare the slopes for each $r_{\delta }$ to the slope for $r_{\delta }=0.1\cdot r_{bas}$ and see how much they deviate from it (Fig. 10*B*,*D*). If, for a given *r_bas_*, increasing *r_δ_* would result in significant change in the regressed *S^s^*, then *G_max_* would be dependent on the stimulus intensity *r_δ_*. We also show the *R*^2^ statistics to confirm the accuracy of the linear approximation (Fig. 10*C*,*E*).

As we observe, for low signal-to-basal ratios ($r_{\delta }/r_{bas}<1$), there is a wide range of rates for which the approximation is good enough, with $|S^{s}$ dev |<1% and *R*^2^ > 99.9%. Specially for low *r_bas_*, the approximation is valid for the whole range of *r_δ_*.

## Discussion

Our results suggest how the activity distribution of a presynaptic population can exploit the nonlinearities of short-term synaptic plasticity and, with that, the theoretical potential of synaptic dynamics to endow a postsynaptic target with the ability to discriminate between weak signals and background activity fluctuations of the same amplitude. Such mechanisms have the advantage of being in-built in synapses, not requiring further recurrent computation or any sort of supervised learning to take place. This feature is likely to be present in different brain regions, e.g. the cerebellum and the hippocampus, and might have critical implications for general information processing in the brain.

### Relevance to specific brain circuits

We have shown that STP can enhance the effective input when (1) stimulus is sparse, temporally bursty and (2) FFE synapses on the principal cells are facilitatory and FFE synapses on local fast-spiking, inhibitory interneurons are depressing. These two conditions are fulfilled in several brain regions. Sparse coding provides many advantages for neural representations (Babadi and Sompolinsky, 2014) and associative learning (Litwin-Kumar et al., 2017). As discussed in the following, a number of experimental studies provide support for sparse coding in several brain regions such as the neocortex, cerebellum and hippocampus.

In the cerebellum, glomeruli in the granular layer actively sparsify the multimodal input from mossy fibers into relatively few simultaneously bursting PFs (Billings et al., 2014) projecting to Purkinje cells (PuC). A single PuC might sample from hundreds of thousands of PFs (Tyrrell and Willshaw, 1992; Ito, 2006). In behaving animals, PF present two stereotypical activity patterns, a noisy basal state with rates lower than 1 Hz during long periods interleaved by short-duration (∼40 ms), high-frequency (usually >100Hz) bursts carrying sensory-motor information (Chadderton et al., 2004; Jörntell and Ekerot, 2006; van Beugen et al., 2013). Given the large number of PFs impinging on to a PuC, the fluctuations in basal rate are as big as the event-related high-frequency bursts. As our analysis shows, if PF synapses were static, the PuC would not be able to discriminate between high-frequency bursts and background fluctuations. However, PF synapses show short-term facilitation when targeting PuC and short-term depression when targeting Basket cells (Atluri and Regehr, 1996; Bao et al., 2010; Blackman et al., 2013; Grangeray-Vilmint et al., 2018). Basket cells perform strong, phasic somatic inhibition to PuCs (Jörntell et al., 2010). This circuit motif closely matches the FF-EI circuit investigated in this work (Fig. 6). Based on these similarities, we argue that one of the functional implications of the specific properties of STP is to enable the PuC to discriminate between information encoded in high-frequency bursts and background activity fluctuations.

In the neocortex, the population code in the layer 2/3 of the somatosensory (De Kock and Sakmann, 2008) and visual cortex of rats (Greenberg et al., 2008) and mice (Rochefort et al., 2009) is believed to be sparse (Petersen and Crochet, 2013), with short-lived bursts (usually <20ms) of high firing rates occurring over low rate spontaneous activity (<0.5Hz). Additionally, it has been recently found that pyramidal cells at layer 2/3 of the mouse somatosensory cortex show short-term facilitation when targeting cells at layers 2/3 and 5 (Lefort and Petersen, 2017). The receptive field properties in the visual cortex are also consistent with the sparse code (Olshausen and Field, 1996). These characteristics suggest that the mechanism to discriminate between weak signals and background fluctuations may also be present in the neocortex. It is believed that such sparse representation at superficial cortical layers indicates strong stimulus selectivity (Petersen and Crochet, 2013), in which case the transient gain, provided by the target-dependent STP configuration of local pyramidal neurons, would be a suitable property for interlayer communication.

In the hippocampus, the Schaffer collaterals bringing signals from CA3 to CA1 operate under low basal firing rates with evoked bursts of high-frequency activity during short periods of time (Schultz and Rolls, 1999). The synapses from pyramidal cells in CA3 to pyramidal cells in CA1 are facilitatory and provide this pathway with extra gain control (Klyachko and Stevens, 2006). Simultaneously, Schaffer collaterals synapses to CA1 stratum radiatum interneurons show larger release probability than to pyramidal neurons (Sun et al., 2005). Therefore, it is likely that this STP-based stimulus/noise discrimination mechanism is also used to improve the transmission of sequential activity from CA3 to CA1.

As we have pointed above, STP configuration in the neocortex, hippocampus and cerebellum are consistent with the configuration that enables the neural networks to take advantage of sparse coding. However, it is important to notice that facilitatory excitatory inputs to other inhibitory cells also exist in the aforementioned circuits. These facilitatory inputs mostly target interneurons that form synapses on distal dendrites. The presence of facilitatory excitatory drive to these classes of inhibitory neurons is, however, unlikely to counteract the distribution-dependent transient gains, because they produce weaker, slower and persistent dendritic inhibition. Consistent with this idea, only parvalbumin-expressing neurons (that synapse on the soma), but not somatostatin-expressing neurons (that synapse on distal dendrites), modulate stimulus response gain (Wilson et al., 2012).

The initial release probability is the most distinguishable STP parameter between Schaffer collaterals synapses onto CA1 pyramidal cells versus CA1 interneurons (Sun et al., 2005). In line with that, our approach predicts that facilitatory mechanisms that steadily increase a low initial release probability during a fast sequence of spikes (low *U*) will have a greater impact on the optimal *OD* and gain amplitude than mechanisms for fast replenishment of resources (low *τ_rec_*). However, the speed of recovery has been shown to be itself an activity-dependent feature (Fuhrmann et al., 2004; Crowley et al., 2007; Valera et al., 2012; Doussau et al., 2017) and this could in principle increase the relevance of *τ_rec_*.

The facilitatory or depressing nature of STP depends on the postsynaptic neuron type (Markram et al., 1998; Reyes et al., 1998; Rozov et al., 2001; Sun et al., 2005; Pelkey and McBain, 2007; Bao et al., 2010; Blackman et al., 2013; Larsen and Sjöström, 2015; Éltes et al., 2017). Target-dependent STP is a strong indication that such short living dynamics are relevant for specific types of information processing in the brain (Middleton et al., 2011; Naud and Sprekeler, 2018). Here, we predict that, when accompanied by specific arrangements of target-dependent STP found experimentally in different brain regions, disynaptic inhibition could further increase the gain of sparse over dense distributions and make it robust even at higher basal activity, when the gain at facilitatory excitation decreases substantially.

Disynaptic inhibition following excitation is a common motif throughout the brain, and different classes of inhibitory neurons are believed to serve distinct computations within their local circuits (Wilson et al., 2012; Jiang et al., 2015). Despite a wide diversity of inhibitory cell types, a classification of FF-I into two main types, perisomatic and dendritic targeting, seems to be coherent with findings throughout the central nervous system. A remarkable attribute of this configuration is the consistency of the short-term dynamics of excitatory synapses across local circuits: depressing to perisomatic and facilitating to dendritic interneurons (Sun et al., 2005; Bao et al., 2010; Blackman et al., 2013; Éltes et al., 2017).

Disynaptic inhibition has been implicated in controlling the precision of a postsynaptic neuron’s response to brief stimulation in the cerebellum (Mittmann et al., 2005; Ito, 2014) and hippocampus (Pouille and Scanziani, 2001). Additionally, the combination of disynaptic inhibition with target-dependent STP has been recently associated with the ability of networks to decode multiplexed neural signals in the cortex (Naud and Sprekeler, 2018). In line with these, our results show a bimodal profile of the readout neuron response to sparse or dense input code. We also demonstrate that, coexisting with the sustained gain during sparse code transmission, in a dense coding scenario, the system produces shorter periods (∼10 ms) of increased (decreased) spike probability right after stimulus onset (offset; Fig. 6*B*, gray line). This results from inhibitory conductances (GABA) which are slower than the excitatory conductances (AMPA). This very short period of firing rate modulation might work as an indication of a widespread basal rate change in the presynaptic population.

### Relationship with previous work

Historically, STP has been prominently explored as a frequency filter which renders an individual neuron as a low-pass filter (when synapses are depressing) or high pass filter (when synapses are facilitatory; Markram et al., 1998; Dittman et al., 2000; Abbott and Regehr, 2004). It has been suggested that under some conditions STD can also interact with subthreshold oscillation to modulate the gain of the neurons (Latorre et al., 2016). With STP the synaptic strength depends on recent history of the incoming spikes in a particular synapse. This automatically makes the downstream neurons more sensitive to transient fluctuations in input spike trains. In most of the previous work this specific property has been exploited for neural coding.

For instance, history dependence of STP means that the effect of serial correlations (that can be seen in the autocorrelogram of spike trains) and spike bursts in the presynaptic activity depends on whether the synapses express STF or STD. Synapses with STD reduce redundancy in the input spike train by reducing the PSPs of spikes that appear with a certain serial correlation or periodicity (Goldman et al., 2002). By contrast, when synapses express STF, they enhance the effect of serial correlations or spike bursts and the readout neuron can function as a burst detector (Lisman, 1997). In fact, both STF and STD can be combined to de-multiplex spike bursts from single spikes (Izhikevich et al., 2003; Middleton et al., 2011; Naud and Sprekeler, 2018). Thus, much emphasis has been put on understanding how STP can be used to extract information encoded in the pattern of spikes of a single input neuron.

Here, we extend this line of work and show how STP may affect the impact of a neuron ensemble on downstream neurons. Previous work has suggested that STP makes a neuron sensitive to transient rate changes. Given this property, when synapses show STD, input correlations can still modulate the neuron output for a wide range of firing rates (de la Rocha and Parga, 2005). Our work reveals a new consequence of the same effect as we show that STP renders the neurons in the brain with input distribution dependent gain, through which sparse-bursty codes could have stronger downstream impact than dense codes with same intensity. Furthermore, we investigate the relative importance of different STP parameters and baseline firing rates for these gains. This novel feature could be a highly valuable asset in low signal-to-noise ratio conditions. Moreover, our results also show how synapses can impose further constrains on the neural code.

### Experimental verification of model predictions

Experimentally, these results can be tested by measuring the distribution of evoked firing rates of the neurons and STP properties of the synapses in the same brain area. Recent technological advances in stimulation systems, allowing for submillisecond manipulation of single and multiple cells spike activity, might soon provide means for fine control of population spike codes in intact tissues (Shemesh et al., 2017). These, together with refined methods for single cell resolution imaging of entire populations (Xu et al., 2017; Weisenburger and Vaziri, 2018), may also allow for scrutinizing the extent of which the proposed synaptic mechanisms for distribution-dependent gain are present in neural networks. Our prediction about the role of background activity in determining the gain of the sparse or dense codes can be tested by changing the overall background activity using chemogenetic techniques.

### Limitations and possible extensions

Here, we made several simplifications and assumptions to reveal that STP of synapses has important consequences for neural coding. Relaxing each of these simplifications and assumptions may affect our conclusions in certain conditions and should be investigated in further studies. In the following we briefly discuss a few crucial simplifications and how they might affect our results.

Our analyses considered the presynaptic activity to be comprised of independent Poisson processes, that is, whenever we choose a set of *N_ext_* presynaptic units to increase their firing rates, we choose them randomly. Because STP is a synapse specific property, input cross-correlation will not affect *PRR* and *Q* and therefore, the presynaptic gain. However, it is well known that input correlation can change the gain of a neuron (Kuhn et al., 2003; de la Rocha and Parga, 2005).

It is conceivable that in some conditions, input correlations can potentially neutralize the advantage of sparse codes over dense codes. The readout neuron fluctuations (and therefore, their output firing rate) are dependent on the input correlation. For the same amount of pairwise correlation, the size of fluctuations in the readout neurons is directly proportional to the number of signal-carrying units (*N_ext_*). A larger *N_ext_* (dense distribution) will elicit larger fluctuations than a smaller *N_ext_* (sparse distribution). This is because for a larger *N_ext_* more input spikes can occur together in the same time bin. Thus, input correlations may amplify the downstream impact of dense input distributions more than the sparse input distributions. The size of this effect of correlations depends on the number of inputs (*N_ext_*) and the amount of correlations (both pairwise and higher-order). However, because cortical activity is weakly correlated (Ecker et al., 2010) such an effect of correlation may not be enough to completely neutralize the advantage of sparse distribution over dense distribution.

We also did not study the effect of spatial location of synapses in transferring the effect of sparse codes over dense codes to the readout neurons. There are at least two possibilities in which dendritic locations of the synapses may weaken the advantage of a sparse input distributions over a dense input distributions. First, when synaptic strength decreases as a function of distance from the soma: it is possible that in the sparse input distribution case, the synapses bringing the information are located far away from the soma while for dense input distribution at least some inputs will be closer to the soma. Therefore, even if on the presynaptic side, a sparse input distribution generates stronger outputs than a dense input distribution, its effect on the postsynaptic neurons may be weakened because of weaker synapses. However, even in this case, because of dendritic nonlinearities and Na^+^/Ca2+ spikes (Larkum et al., 2009), distally located sparse distribution may still have a stronger response than proximally located dense input distributions. Second, the effect of synapses on certain dendrites is cancelled by strategically placed inhibitory synapses (Gidon and Segev, 2012). It is possible that a sparse distribution (because of fewer synapses) may get cancelled or weakened by strategically placed inhibition. The effect of such inhibition will indeed be weaker on dense input distributions as many more synapses will carry the input information. Thus, for sparse input distributions, their location on the neuron may be an important factor. A proper treatment of this question requires the knowledge of, e.g., neuron morphology, distribution of inhibition, and dendritic non-linearities, and should addressed in a separate study.

We also assumed that all the synaptic weights are sampled from the same Gaussian distribution as our goal was to consider a naive situation in which weights have not been “trained” for any specific task. Having different synaptic weight distributions may affect the value of the gains, especially when synaptic weights and input are associated (stimulus-specific tuning). Such different distributions may arise because of supervise/unsupervised learning. A systematic study of a network with stimulus-specific tuning of synaptic weights raises several pertinent questions and should be investigated in a separate study.

The transient enhancement or depression of synaptic efficacy by presynaptic mechanisms consists of many independent processes (Zucker and Regehr, 2002). The TM model is a tractable and intuitive way to account for these two phenomena of interest, but this parsimony comes at the cost of biophysical simplifications. For example, it assumes the space of available resources is a continuum (0 < *x *<* *1) as opposed to the known discrete nature of transmitter-carrying vesicles. However, we argue that when modeling a large number of simultaneously active synapses, the variable of interest (population *PRR*) can be approximated by a continuous variable. The nonuniform amount of transmitters per vesicle might further justify this assumption.

Detailed STP models that try to account for specific intracellular mechanisms (Dittman et al., 2000), and stochasticity of the release process (Sun et al., 2005; Kandaswamy et al., 2010) have been proposed in the literature. We argue that, with more complex models of STP, our results might change quantitatively but the qualitative outcome of our analysis would remain: that presynaptic short-term facilitation (depression) yields a substantial positive (negative) gain to sparse over dense population codes. Nevertheless, it would be interesting to see how the gain and optimal rate predictions may be shaped by more detailed models.

Our analyses do not account for use-dependent recovery time, changes in the readily releasable pool size (Kaeser and Regehr, 2017) or vesicles properties heterogeneity. The effects of postsynaptic receptor desensitization and neurotransmitter release inhibition by retrograde messengers (Brown et al., 2003) are likely to decrease the estimated gain by counteracting facilitation. Another interesting extension could be used to further investigate the effects of input STP heterogeneity at compartment-dependent input using multi-compartment neuron models (Vetter et al., 2001; Grillo et al., 2018).

If the same patterns of bursts tend to happen repeatedly (e.g., PFs in cerebellum during continuously repetitive movement), there might be an optimal interburst interval (*IBI^opt^*) for which, if bursts happen faster than *IBI^opt^*, the signal would be compromised (because of slow vesicles recovery time) and if bursts happen separated by intervals longer then *IBI^opt^* no extra gain will happen. Experimental evidence points to the importance of resonance in the band oscillations (5.0∼10.0 Hz,interburst interval: 100∼200 ms IBI) for cortical-cerebellar drive (Gandolfi et al., 2013; Chen et al., 2016) and for hippocampus (Buzsáki, 2002). In these cases, the slower interaction between different pools of vesicles (Rizzoli and Betz, 2005) are likely to play a role in information transfer. Augmentation, a form of transient synaptic enhancement that can last for seconds, is also likely to play a role in these cases (Kandaswamy et al., 2010; Deng and Klyachko, 2011).

**Table 1.** List of recurrent symbols

Symbol	Description	Equation
*U*	Facilitation factor	1
*τ_rec_*	Recovery time constant	1
*τ_f_*	Facilitation time constant	1
*r*	Firing rate of a single synapse	2
*PRR*	Proportion of released resources	2
*Q*	Total *PRR* (integrated over a time interval)	3
*T_s_*	Duration of the stimulus interval	3
*N*	Number of presynaptic neurons	4
*G*	Gain of any given distribution of presynaptic activity over the maximally distributed presynaptic activity ($N_{ext}=N$)	6
*OD*	Optimal distribution of the presynaptic activity	7
*s*1, *s*2	Synaptic type 1, synaptic type 2	12
*r_bas_*	Individual basal firing rate	
*r_ext_*	Individual increase in firing rate	
$r_{\delta }$	Individual increase in firing rate when $N_{ext}=N$	
*r_opt_*	Optimal individual increase in firing rate when *N_ext_* = *N_opt_*	
*R_bas_*	Total population basal firing rate	
*R_ext_*	Total population increase in firing rate	
*X^s^*	A *s* superscript indicates a quantity for a single synapse	
*X^p^*	A *p* superscript indicates a quantity for a population of synapses	
*X_bas_*	A *bas* subscript indicates a quantity for basal level activity	
*X_ext_*	A *ext* subscript indicates a quantity for extra activity	
$X_{\delta }$	A *δ* subscript indicates a quantity for the maximally distributed presynaptic activity ($N_{ext}=N$)	
*X_opt_*	A *opt* subscript indicates a quantity for the optimally distributed presynaptic activity (*N_ext_* = *N_opt_*)	

## Synthesis

Reviewing Editor: Liset Menendez de la Prida, Instituto Cajal CSIC Decisions are customarily a result of the Reviewing Editor and the peer reviewers coming together and discussing their recommendations until a consensus is reached. When revisions are invited, a fact-based synthesis statement explaining their decision and outlining what is needed to prepare a revision will be listed below. The following reviewer(s) agreed to reveal their identity: Pablo Varona.

Your ms was evaluated by two reviewers and myself. We all agree the paper has some merits but it requires some additional work before a decision can be made.

1- We feel the ms structure should be optimized. In the current version, figures do not follow the standard order and fundamental aspects of the model are introduced later than others. The paper would benefit from a better organization especially at the beginning, so that the modeling approach is more accessible to the reader.

2- While you devoted a section to previous work, we feel you are missing some historical perspective on the subject. There are previous reports on distinct coding schemes emerging by facilitating and depressing synapses. You need to revise bibliography more carefully.

3- Both reviewers agree that a limitation of your model is that it considers purely passive IF neurons that only dependent on the synaptic conductances. In particular, your study fails to address how the sensitivity to input distribution (and gain) is affected by interactions between presynaptic STP mechanisms and postsynaptic filtering mechanisms. This may include, for instance effects of nonlinearities such as those introduced by NMDA receptors or HCN channels, the effect of subthreshold membrane potential oscillations or active somatodendritic conductances (see for instance Latorre et al, PLoS ONE 2006 https://doi.org/10.1371/journal.pone.0145830; de la Rocha and Parga JN 2005 https://doi.org/10.1523/JNEUROSCI.0631-05.2005). While we feel you can acknowledge this in the section on Limitations, we all agree your work will benefit from some additional modeling/analysis to better extrapolate your conclusions. We leave to you the decision on what is more convenient to evaluate but we would like to see this additional work implemented somehow.

4- There are some open questions you should further consider. We compile all of them here. While we agree each of them can be the matter of a new work, we think they are useful for you to consider in relation with the previous point 3. Open questions: Does the model display the same results for less populated inputs channels? Why the effect of asymmetry in the synaptic weights was not studied in the simulations? How changes in the expression of postsynaptic receptors and ion-channels (such as NMDA receptors and HCN channels) interplay with STP induced non-linear synaptic dynamics? How does synapse location (i.e. soomatodendritic) interact with STP gain and input distribution preference shaped by facilitation and depression?

5- Please, report your model code in a public repository.

6- The visual abstract is missing

## Author Response

Dear Dr. de la Prida

Thank you for giving us an opportunity to revise the manuscript. The reviewers made many insightful remarks and provided us with crucial feedback. We thank them also. We also apologize for the delay in revising the manuscript.

We have now revised the manuscript according to the feedback from the reviewers. The changes in the manuscript are marked in blue.

In the following we have provided point-by-point replies to show how we have addressed all the concerns raised by the reviewers. Our reply is marked in normal font and reviewers concerns are in boldface.

Best regards

Authors

1- We feel the ms structure should be optimized. In the current version, figures do not follow the standard order and fundamental aspects of the model are introduced later than others. The paper would benefit from a better organization especially at the beginning, so that the modeling approach is more accessible to the reader.

We agree that in the Methods section figures are not cited in a numerical order. But believe that it is helpful to assist the reader to locate where results corresponding to a certain method can be found. As such we think that the Methods are also presented in a logical fashion. In the Results section all the figures are presented and discussed in their numerical order. So unless the reviewers are thinking of a particular reordering, we would like to keep the flow of the manuscript the same. Now with further clarifications we think that the manuscript has become more accessible. One of things that might have made the manuscript a bit cumbersome is the number of symbols we have used. To further assist the reader we have extended Table 1 with symbols corresponding to several key variables that were previously missing from the table of symbols.

2- While you devoted a section to previous work, we feel you are missing some historical perspective on the subject. There are previous reports on distinct coding schemes emerging by facilitating and depressing synapses. You need to revise bibliography more carefully.

We agree with the reviewers that some historical perspective on computational properties of short-term synaptic dynamics was lacking. We extended the subsection Relationship with previous works. [See lines 696-718]

3- Both reviewers agree that a limitation of your model is that it considers purely passive IF neurons that only dependent on the synaptic conductances. In particular, your study fails to address how the sensitivity to input distribution (and gain) is affected by interactions between presynaptic STP mechanisms and postsynaptic filtering mechanisms. This may include, for instance effects of nonlinearities such as those introduced by NMDA receptors or HCN channels, the effect of subthreshold membrane potential oscillations or active somatodendritic conductances (see for instance Latorre et al, PLoS ONE 2006 https://doi.org/10.1371/journal.pone.0145830; de la Rocha and Parga JN 2005 https://doi.org/10.1523/JNEUROSCI.0631-05.2005). While we feel you can acknowledge this in the section on Limitations, we all agree your work will benefit from some additional modeling/analysis to better extrapolate your conclusions. We leave to you the decision on what is more convenient to evaluate but we would like to see this additional work implemented somehow.

We agree with the reviewers that we should have discussed how non-linearities in the postsynaptic neuron (e.g. HCN channels and NMDA receptors) may affect the distribution-dependent presynaptic gains. In the following we discuss the effects of synaptic and dendritic non-linearities and how we have modified the manuscript.

Dendritic/postsynaptic non-linearities: A study of the effects of dendritic/synaptic non-linearities is a highly non-trivial task, but we have investigated the effect of non-linearities introduced by including NMDA receptors. To this end, we simulated a two-compartment neuron model (Figure 1 below) -- in a single compartment model we cannot see the effects of non-linear change due to the removal of the magnesium (Mg2+)

block. Indeed with NMDA receptors the neuron output as a function of synaptic input magnitude (transfer function) shows a threshold-like non-linearity.

Our main claim is that given STP, sparse and dense distribution of inputs will result in different effective input (QS1, QS2) to the postsynaptic side. When we consider the case that dense input patterns elicit weaker response then sparse input patterns (facilitatory synapses), three scenarios can arise on the postsynaptic side.

1. The effective inputs for sparse and dense inputs are too weak to activate the NMDA receptors: This would imply that the two inputs will be transformed into spikes using the same linear transfer function of the neuron (blue curve in the figure 1). The two inputs corresponding to dense and sparse are marked as points ‘1’ and ‘2’ in the figure. In this case, the gain in output rate between sparse and dense inputs will be exclusively due to the presynaptic gain.

2. The effective inputs for sparse inputs are strong enough to activate NMDA receptors, while dense inputs are not: This would be caused by the extra accumulation of neurotransmitters in specific sites due to bursts of the sparse distribution, which a dense distribution would not attain. In this scenario, dense inputs will still be transformed to spikes by the linear transfer function of the neuron (blue curve in the figure 1) while the sparse inputs will be transformed to spikes by the non-linear transfer function (red curve in Fig 1). The two inputs corresponding to dense and sparse are marked as points ‘1’ and ‘4’ in the figure. In this case, the gain in output rate between sparse and dense inputs will be further magnified by the NMDA nonlinearity.

3. The effective inputs for sparse inputs and dense inputs are both strong enough to activate NMDA receptors: this would imply that both inputs will be transformed to spikes using the non-linear transfer function (red curve in Fig 1). The two inputs corresponding to dense and sparse are marked as points ‘3’ and ‘4’ in the figure. Similar to the first scenario, sparse and dense inputs will elicit different numbers of output spikes solely due to the presynaptic gains.

These results show that non-linearities induced by NMDA receptors may not obscure the effects of presynaptic distribution-dependent gains, and may only further enhance these.

These simulations also give us insights into how other non-linearities might affect our main result. As long as the neuron transfer function is not flat for a range of inputs, i.e. inputs do not drive the neuron into its saturation regime, we will see the effect of presynaptic distribution-dependent gains -- the magnitude of the effect will depend on the slope of the neuron transfer function.

In the manuscript we have not included this figure because this will require a long description of the modelling of a two-compartment model. Moreover, we have chosen the parameters which seemed reasonable i.e. we obtained reasonable firing rates and the model behaved identically with or without NMDA receptors for weak inputs. Finally, if we have to add this model in the manuscript it would be necessary to do a proper analysis of when and how much non-linearity is seen -- which we think is beyond the scope of this manuscript.

Therefore, we have used a schematic of the transfer function shown in the figure 1 (see below) to make the arguments in the manuscript in a more general way. We hope that the reviewers will agree with us on this choice. Based on these results and insights, we have added a new subsection From presynaptic gains to postsynaptic rate changes in the discussion section to discuss the issue of non-linear synaptic integration in the postsynaptic neuron [See lines 552-581].

Figure 1: We simulated a two compartment neuron model. The two compartments were coupled using a resistance. Synapses were placed on the non-spiking (dendritic) compartment. The synapses were modelled as either AMPA type or combined AMPA and NMDA type. NMDA and AMPA components had equal area. To mimic different effective input (==QS1, QS2) we varied the strength of the input and measured the number of spikes elicited in a short time window of 100ms. The neuron transfer function in the absence of NMDA synapses is shown in the blue curve. Individual pale blue lines show individual trials and the dark blue curve is an average of

30 independent trials. The neuron transfer function with NMDA synapses is shown in the red curve. Individual pale red lines show individual trials and the dark red curve is an average of 30 independent trials. Beyond a certain input, Mg block was released and the dendritic compartment observed a strong depolarization, therefore the slope of the neuron transfer was higher for a small range of inputs. The parameters of the model were tuned in such a way that for weak inputs, the neuron responded with similar inputs in both cases (with and without NMDA).

Effect of correlations: The study by la Rocha and Praga 2005 investigated the effect of correlations on neuron transfer function. But that analysis was done for the steady state of neurons i.e. inputs were stationary and after some time PSP reached their steady state values. So that part does not relate to our work directly.

However, this made us think about the role of input correlations. Indeed correlations in the input could potentially reduce the gain of sparse input patterns. If there are input correlations, in a dense input pattern, more inputs can spike together. By contrast, in sparse input patterns, because only a few neurons carry spiks, only a few neurons can spike together. That is, for dense inputs, if inputs are highly correlated, fluctuations in the input current can be larger than those observed for sparse inputs with similar amount of pairwise correlations. This is a possibility by which input correlation could amplify the effect of dense inputs and such amplification would be smaller for the sparse inputs. The size of this effect of correlations depends on the number of inputs and amount of correlations (both pairwise and higher-order). And significant effects can only be seen for highly correlated input patterns. However, most experimental evidence suggests that stimulus-evoked activity often has rather low correlations (e.g. see Ecker et al. 2010). We have added new text to discuss this issues [See lines 741-757]

Effect of subthreshold oscillations: The results of Lattore et al. 2006 will indeed be relevant for more detailed accounts of neurons operating under subthreshold oscillatory regimes. We believe that some of these results could be intuitively explained by a similar rationale of qualitatively different transfer functions, as developed on our arguments above and also on the subsection From presynaptic gains to postsynaptic rate changes. In short, the combined effects of input-structure and STP on the readout neuron firing, operating through changes in

subthreshold oscillations, can be understood as changes in the neuron’s effective transfer function, see added section From presynaptic gains to postsynaptic rate changes.

4- There are some open questions you should further consider. We compile all of them here. While we agree each of them can be the matter of a new work, we think they are useful for you to consider in relation with the previous point 3. Open questions:

4a: Does the model display the same results for less populated inputs channels? Why the effect of asymmetry in the synaptic weights was not studied in the simulations?

We thank the reviewers for raising these questions. We missed on discussing the role of population size. We have now revised the text [See lines 105-113 and 329-341] to elaborate on this. Briefly, our results will hold as long as N is larger than N_opt.

We have shown that given the STP parameters, there is an optimum firing rate r_opt through which signal carrying neurons should operate to maximize gain (Figure 2F), this optimum rate is independent of population size N. For a given incoming stimulus rate R_ext, it is optimal to distribute the spikes over N_opt = R_ext/r_opt input channels (e.g. Figure 2F). If N

<= N_opt, then we cannot talk about sparseness. The argument for sparseness arises when

N >> N_opt.

When N>> N_opt, if we change the population size N while keeping all other parameters constant, OD will monotonically decrease as a function of N. However, it is important to note that if we change N we should also change R_ext otherwise the signal-to-noise ratio will change. Signal to noise ratio here is defined as R_{ext}/R_{bas} (for the activity is Poissonian). If we keep r_bas constant and change N, then R_{bas} = Nxr_bas will also change. Therefore, we need to scale R_ext accordingly. That is, without scaling R_ext we cannot meaningfully compare low and high N scenarios. Therefore, to compare different N, we should scale the total input rate R_ext with N. And with this choice OD is independent of N (eq 7).

Asymmetry in synaptic weights: I meant heterogeneity in synaptic weights, not asymmetry. [This question was clarified with the editor. In the following lines we have appended the clarification.] Weights of dynamic synapses do affect the output when interacting with the intrinsic dynamics of the postsynaptic neurons beyond gain changes (see references cited in my report). This comment is oriented to assess the robustness of the phenomena under the more realistic case of heterogeneous weights. In the discussion you seem to suggest this kind of study for a future paper, but I find it easy to test in your current model. You decide whether you want to include this or not. In my opinion, it would improve the impact of your paper.

The parameters values for every input unit in our neuronal network simulations were randomly sampled from Gaussian distributions, including the synaptic weights [See lines

446-448].

However, it is possible that input synaptic weights follow a different synaptic weight distributions (e.g. that emerge due to some supervised/unsupervised learning). Such distributions may affect the value of the gains, especially when weights and input are associated (stimulus-specific tuning). Indeed, if we are allowed to change each synapse we can find appropriate weight distributions that will magnify the effects of either sparse or dense or both types of input distributions. Our goal here was to consider a naive situation in

which weights were not ‘trained’ for any specific task. A systematic study of a network with stimulus-specific tuning of synaptic weights raises several pertinent questions and should be investigated in a separate study. We have clarified in the text [See lines 776-782].

4b: How changes in the expression of postsynaptic receptors and ion-channels (such as NMDA receptors and HCN channels) interplay with STP induced non-linear synaptic dynamics?

We have addressed this question above in response to question 3 (see figure 1).

4c: How does synapse location (i.e. soomatodendritic) interact with STP gain and input distribution preference shaped by facilitation and depression?

This is an interesting question. We can think of two possibilities in which dendritic locations of the synapses may neutralize the advantage of a sparse input distribution over a dense distribution (with facilitatory synapses). First, when synaptic strength (measured as PSP amplitude in the soma) decreases as a function of distance from the soma: It is possible that in the sparse input pattern case, the bursting synapses are mostly located far away from the soma while, for dense input distribution at least some inputs will be close to the soma. Therefore, it is possible that sparse distribution may have the same or even weaker effect on the soma than dense input patterns. However, even in this case, far away synapses may be rescued by dendritic nonlinearities (as shown in Figure 1 above) and calcium spikes etc. Second, the effect of synapses on a certain dendrites is cancelled by strategically placed inhibitory synapses (e.g. Gidon and Segev 2012): In this scenario again, since sparse input is carried by fewer synapses, it is possible that they may get cancelled or weakened by strategically placed inhibition. The effect of such inhibition will indeed be weaker on dense inputs as many more synapses will carry the input information.

Thus, for sparse inputs, their location on the neuron may indeed be an important factor. A proper treatment of this question requires the knowledge of neuron morphology, distribution of inhibition, dendritic non-linearities etc. So we cannot expand the current manuscript to address this. But we have discussed this question in the discussion and it makes for a very interesting next study [See lines 758-775].

5- Please, report your model code in a public repository.

We will have the code on Github but we were asked to remove the link for double-blind peer-review.

6- The visual abstract is missing

We have uploaded a figure with the visual abstract.
