# Erratum: Tauffer and Kumar, “Short-Term Synaptic Plasticity Makes Neurons Sensitive to the Distribution of Presynaptic Population Firing Rates”

**DOI:** 10.1523/ENEURO.0571-24.2024

**Published:** 2025-01-17

In the article “Short-Term Synaptic Plasticity Makes Neurons Sensitive to the Distribution of Presynaptic Population Firing Rates,” by Luiz Tauffer and Arvind Kumar, which was published online on February 12, 2021, an affiliation was accidentally omitted for Arvind Kumar. The missing affiliation is SciLifeLab, Stockholm, Sweden. The corrected author affiliations are listed below.

Luiz Tauffer^1,2^ and Arvind Kumar^1,2,3^

^1^Department of Computational Science and Technology, School of Computer Science and Communication, KTH Royal Institute of Technology, Stockholm 11428, Sweden, ^2^Bernstein Center Freiburg, University of Freiburg, Freiburg im Breisgau 79104, Germany, and ^3^SciLifeLab, Stockholm, Sweden

